# Studies toward the First Stereoselective Total Synthesis of (±)-Quinolizidine 195C and Other Transformations

**DOI:** 10.3390/molecules18078243

**Published:** 2013-07-12

**Authors:** Shang-Shing P. Chou, Jhih-Liang Huang

**Affiliations:** Department of Chemistry, Fu Jen Catholic University, New Taipei City 24205, Taiwan; E-Mail: nopim520@gmail.com

**Keywords:** aza-Diels-Alder reaction, intramolecular aza-Michael reaction, quinolizidines, quinolizidine 195C

## Abstract

Starting from a thio-substituted 4-quinolizidinone, a series of C-6 alkylated derivatives with a *trans* C-6, C-9a relationship was synthesized. Further transformations led to the first stereoselective total synthesis of the structure proposed for (±)-quinolizidine 195C, the major alkaloid isolated from the skin extracts of the Madagascan frog *Mantella betsileo*. Since the spectral data of the synthetic and natural products differed significantly, the true structure of (±)-quinolizidine 195C remains uncertain.

## 1. Introduction

**Scheme 1:**
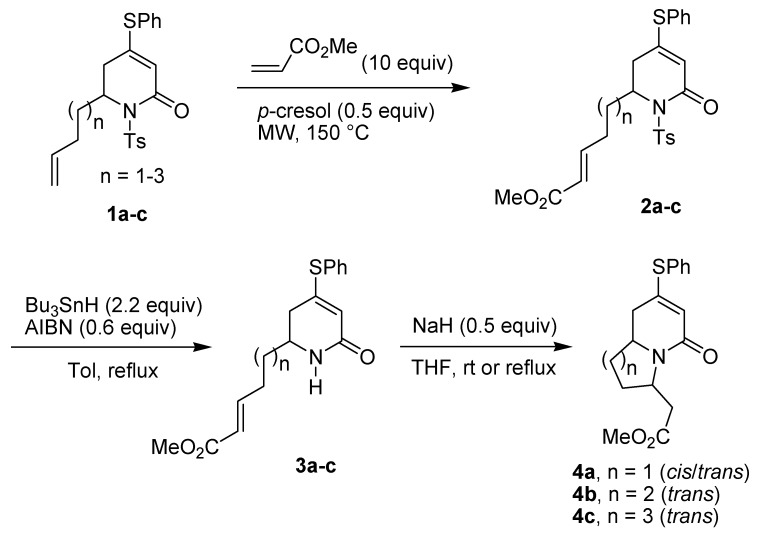
Cross-metathesis and aza-Michael reaction.

The piperidine ring is one of the most abundant molecular fragments in both natural and synthetic compounds displaying various biological activities [1,2,3,4]. Among the piperidine natural products are the bicyclic indolizidines and quinolizidines [5,6,7,8,9,10,11,12,13,14,15,16]. Imino-Diels-Alder reactions are very useful for the synthesis of tetrahydropyridines [17,18,19]. We have previously developed a new imino-Diels-Alder reaction of thio-substituted 3-sulfolenes with *p*-toluenesulfonyl isocyanate (PTSI) to synthesize piperidine derivatives [20,21], and have used this method to prepare some indolizidines and quinolizidines [22,23,24,25,26,27,28,29,30,31,32]. We have recently reported the use of cross metathesis (CM) to transform the terminal alkenes **1a**–**c** into the α,β-unsaturated esters **2a**–**c**, and after detosylation the resulting amides **3a**–**c** can undergo the intramolecular aza-Michael reaction to give sulfur-substituted bicyclic compounds **4a**–**c** (Scheme 1) [33].

**Figure 1:**
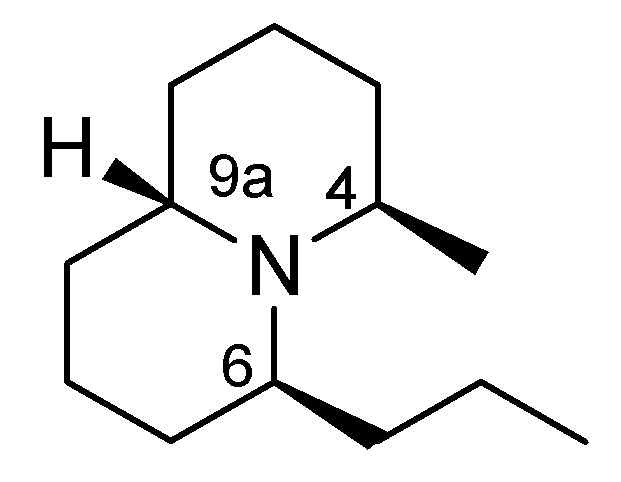
Proposed structure of quinolizidine 195C.

Herein a new method for the synthesis of C-6 alkylated quinolizidines with a *trans*-C-6, C-9a relationship is reported, and the *trans*-compound **4b** could be used for the preparation of quinolizidine 195C (Figure 1), which was isolated as a major alkaloid from the skin extracts of the Madagascan frog *Mantella betsileo* [34,35]. The proposed structure of quinolizidine 195C was based only on its mass spectral and FTIR data. There has been no synthesis of quinolizidine 195C itself, and only that of (±)-quinolizidine 9a-*epi*-195C was reported [36]. We also reported recently the synthesis of (±)-quinolizidine 4-*epi*-195C and (±)-quinolizidine 9a-*epi*-195C [37]. We hoped to achieve the synthesis of quinolizidine 195C and to confirm its structure by providing more spectral data.

## 2. Results and Discussion

**Scheme 2:**
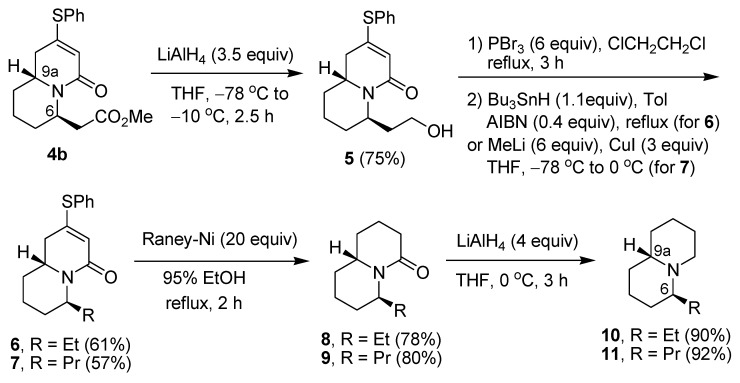
Synthesis of compounds **8**–**11**.

Reduction of compound **4b** [33] with LiAlH_4_ in THF at low temperature gave the primary alcohol **5** (Scheme 2). Further treatment with PBr_3_ provided the expected bromide, but since the bromide was rather unstable, we directly carried out the debromination with Bu_3_SnH/AIBN to give the ethyl-substituted product **6**. If the bromide was treated with Me_2_CuLi, the propyl-substituted product **7** was obtained. Further reactions of compounds **6** and **7** with Raney nickel cleaved the C-S bond and also reduced the C=C bond to give the products **8** and **9**, respectively. Compounds **8** and **9** were then reduced with lithium aluminum hydride in THF at 0 °C to give the corresponding quinolizidines **10** and **11**. Since compound **4b** has been previously established to have a *trans-*C-6, C-9a relationship [33], the compounds **10** and **11** should retain the same configuration. The ^1^H- and ^13^C-NMR spectral data of compound **11** however were significantly different from those of the *cis* isomer reported in the literature [36].

We then studied the conversion of compound **9** to quinolizidine 195C. Unfortunately, various reaction conditions of compound **9** with a methyl nucleophile, followed by treatment with acetic acid and NaBH_4_/MeOH all failed, as shown in Table 1. Reaction of compound **9** with methylmagnesium bromide at 65 °C gave only the recovered starting material (entry 1). The reaction with methyllithium at room temperature gave the same result (entry 2). Increasing the reaction temperature to 50 °C, however, gave an unidentified mixture of products (entry 3). We suspect that the axial propyl group of compound **9** at C-6 hinders the reaction of the nucleophile with the C=O group.

**Table 1:** Reactions of compound **9** with a methyl nucleophile. 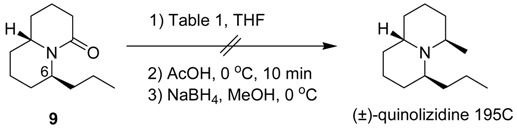

Entry	Reaction Conditions	Results
1	MeMgBr (4 equiv), 65 °C, 3 h	NR ^a^
2	MeLi (5 equiv), rt, 5 h	NR ^a^
3	MeLi (5 equiv), 50 °C, 5 h	ND ^b^

^a^ No reaction was observed; ^b^ An unidentified mixture of products was obtained.

We then decided to react compound **7** with the carbon nucleophile, because the more planar structure of compound **7** should offer less steric hindrance (Table 2). Treatment of compound **7** with methylmagnesium bromide, followed by acidification with acetic acid and reduction with NaBH_4_ at 0 °C (entry 1) gave the 1,2-addition products **12** and **13** (in a ratio of 2:1), and some undesired 1,4-substitution product **14**. Since compound **12** has the correct stereochemistry for the conversion to quinolizidine 195C, we tried to increase the **12**/**13** ratio. When a bulkier reducing agent, NaB(OAc)_3_H, was used at 0 °C (entry 2), the ratio of **12**/**13** remained the same at 2:1. If the reduction with NaB(OAc)_3_H was carried out at room temperature (entry 3), the ratio of **12**/**13** was reduced to 1.5:1. In contrast, carrying out the reaction at −50 °C for 2 h and then slowly warming to 0 °C (entry 4) afforded the products **12** and **13** in a higher ratio of 4:1. From these results it seems that compound **12** is the kinetic product, which is more favored at lower temperature. However, use of a more bulky and less reactive reductant like NaB(OAc)_3_H or NaBH_3_CN at low temperature gave only the 1,4-substitution product **14** (entries 5 and 6). This could be attributed to a slower rate of reduction of the iminium ion intermediate at low temperature by the less reactive reductants. The unreacted iminium ion was then hydrolyzed during aqueous work-up, or was lost in the aqueous solution.

**Table 2:** Reactions of compound **7** with MeMgBr followed by reduction. 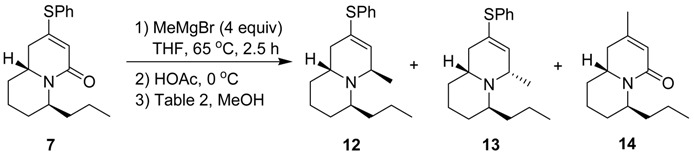

Entry	Reaction Conditions	Products (%Yield) ^a^	Ratio of12/13 ^b^
1	NaBH_4_, 0 °C, 2.5 h	**12** (27), **13** (13), **14** (31)	2:1
2	NaB(OAc)_3_H, 0 °C, 2 h	**12** (29), **13** (14), **14** (27)	2:1
3	NaB(OAc)_3_H, rt, 2 h	**12**/**13** (33%), ^c^ **14** (30)	1.5:1
4	NaBH_4_, −50 °C, 2.5 h, then to 0 °C, 2 h	**12** (34), **13** (8), **14** (38)	4:1
5	NaB(OAc)_3_H, −50 °C to 0 °C, 2 h	**14** (34)	-
6	NaBH_3_CN, −50 °C to 0 °C, 2 h	**14** (36)	-

^a^ Isolated yield of the purified products; ^b^ The ratio of **12**/**13** was determined from the ^1^H-NMR of crude reactions mixtures; ^c^ The mixture of compounds **12** and **13** was not separated.

**Figure 2:**
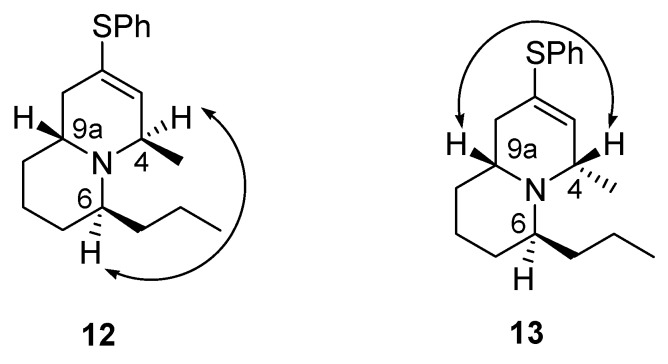
NOESY correlations of compounds **12** and **13**.

**Scheme 3:**
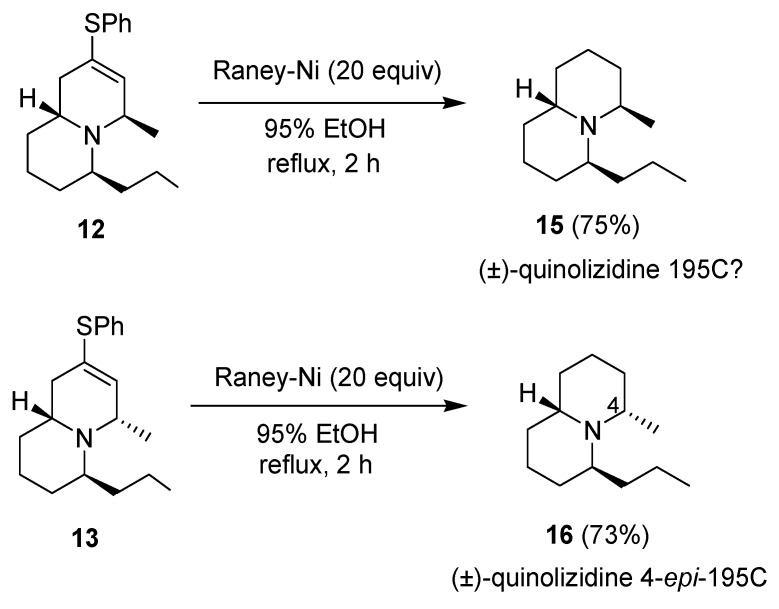
Preparation of compounds **15** and **16**.

The stereochemistry of compounds **12** and **13** was determined from their NOESY spectra. Compound **12** shows cross signals between the hydrogens at C_4_ and C_6_, but no cross signals between the hydrogens at C_6_ and C_9a_. On the other hand, compound **13** shows cross signals between the hydrogens at C_4_ and C_9a_, but no cross signals between the hydrogens at C_4_ and C_6_ (Figure 2). Further treatment of compounds **12** and **13** with Raney nickel in refluxing 95% EtOH gave compounds **15** and **16**, respectively (Scheme 3). The spectral data of compound **16** were identical with what we had earlier reported for (±)-quinolizidine 4-*epi*-195C [37]. On the other hand, the three most downfield CHN protons of compound **15** at *δ* 3.0–3.2 are quite different from the partial ^1^H-NMR data (*δ* 3.7–4.0) of (±)-quinolizidine 195C descried in the literature [34]. Compound **15** exhibits a weak Bohlmann band at 2795 cm^–1^ whereas the literature data for (±)-quinolizidine 195C was at 2811 cm^–1^ [34]. Thus, since we are quite confident about the structure of compound **15** based on all the spectral data and related chemical reactions, its identity with the true structure of (±)-quinolizidine 195C remains uncertain.

**Scheme 4:**
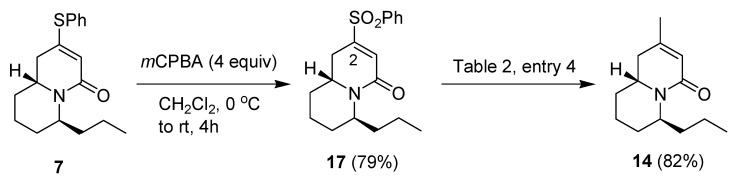
Preparation of compound **17** and reaction with a nucleophile.

Since the reaction of compound **7** with MeMgBr under various conditions of Table 2 all gave significant amounts of the 1,4-substitution product **14**, we hoped that the conversion of the phenylthio group of compound **7** first to the sulfone **17** would disfavor the formation of the 1,4-substitution product **14** because of the greater steric hindrance of the phenylsulfonyl group. However, the reaction of compound **17** with methylmagnesium bromide, followed by HOAc and NaBH_4_ gave only the 1,4-substitution product **14** (Scheme 4). 

It seems that the strongly electron-withdrawing phenylsulfonyl group increases the partial positive charge at C-2 significantly and outweighs its steric effect so that only the 1,4-substitution was observed. 

**Scheme 5:**
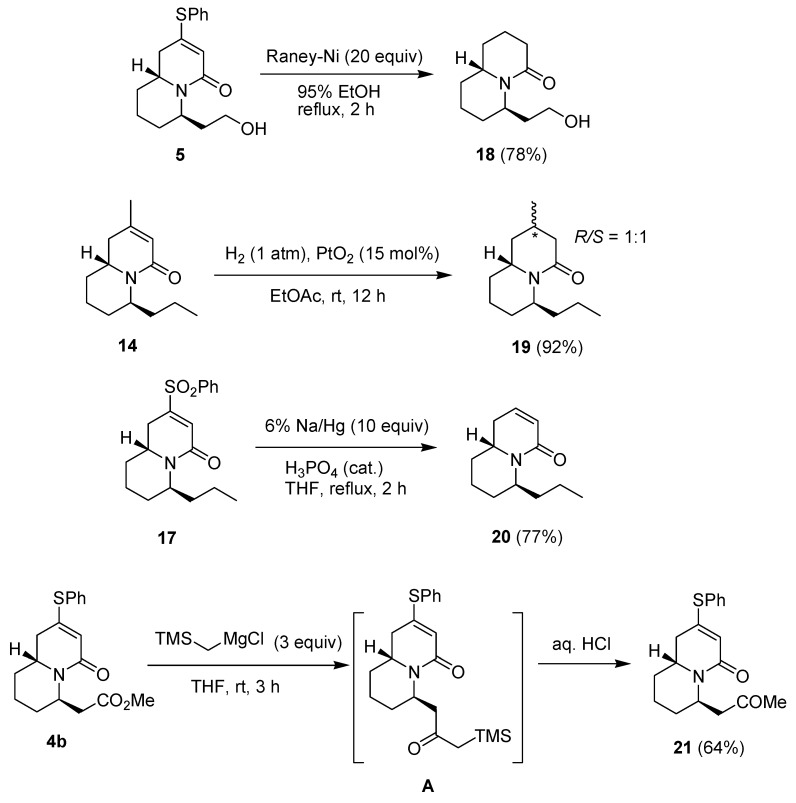
Preparation of compounds **18**−**21**.

We are also interested in studying the biological effects of the quinilizidine derivatives, so we have carried out further synthetic transformations of some of these quinolizidines (Scheme 5). Reaction of compound **5** with Raney nickel in refluxing 95% EtOH gave the product **18**. Under catalytic hydrogenation conditions, compound **14** was converted to an inseparable 1:1 mixture of compounds **19.** Treatment of compound **17** with 6% sodium amalgam in the presence of a small amount of phosphoric acid [38,39] resulted in the selective cleavage of the phenylsulfonyl group to give product **20**. We also found that the reaction of compound **4b** with an excess of (trimethylsilyl)methylmagnesium chloride, followed by treatment with aqueous HCl solution gave the methyl ketone product **21**. Presumably, the reaction proceeds through the single addition/elimination intermediate **A**, which is sterically too hindered to react with another molecule of the Grignard reagent.

## 3. Experimental

### General

Infrared spectra were recorded with a Perkin-Elmer 100 series FTIR spectrometer using the ATR (attenuated total reflectance) mode. ^1^H- and ^13^C-NMR spectra were recorded on a Bruker Avance 300 spectrometer operating at 300 and at 75 MHz, respectively. Chemical shifts (*δ*) are reported in parts per million (ppm) and the coupling constants (*J*) are given in Hertz. High-resolution mass spectra (HRMS) were measured with a Finnigan/Thermo Quest MAT 95XL mass spectrometer, or a Shimadzu LCMS-IT-TOF mass spectrometer for electrospray ionization (ESI) measurements. Flash column chromatographic purifications were performed using Merck 60 H silica gel. The microwave reactions were carried out with a CEM Focused^TM^ Discover-S system.

*Trans-6-(2-hydroxyethyl)-2-(phenylthio)-1,6,7,8,9,9a-hexahydro-4-quinolizinone* (**5**). To a solution of compound **4b** (510 mg, 1.54 mmol) in THF (50 mL) under nitrogen was added dropwise LiAlH_4_ (2.4 M in THF, 2.6 mL, 6.16 mmol) at −78 °C. The mixture was slowly warmed to −10 °C in 2.5 h. The solvent was removed under reduced pressure. Saturated NaHCO_3_ solution was then slowly added. The mixture was extracted three times with ethyl acetate. The organic solution was concentrated under reduced pressure and dried (MgSO_4_) to give the crude product, which was purified by flash chromatography on silica gel prewashed with Et_3_N using ethyl acetate/hexane (1:1) and 5% Et_3_N as eluent to give product **5** (350 mg, 75%). Colorless oil; ^1^H-NMR (CDCl_3_) δ 7.51–7.40 (5H, m), 5.31 (1H, d, *J* = 1.8 Hz), 4.77–4.73 (1H, m), 3.96 (1H, OH), 3.60–3.49 (2H, m), 3.30–3.22 (1H, m), 2.57–2.38 (2H, m), 1.89–1.38 (8H, m); ^13^C-NMR (CDCl_3_) δ 164.8, 151.8, 135.1, 129.8, 129.7, 127.9, 114.5, 58.2, 49.0, 45.1, 35.3, 33.4, 33.2, 28.5, 18.1; IR (neat) 3404, 3054, 2943, 2873, 1675, 1596, 1436, 1335, 1266, 1068, 864, 705 cm^−1^; EI-MS (rel. intensity) *m/z* 303 (M^+^, 8), 272 (27), 259 (44), 258 (100), 231 (29), 229 (53), 119 (24), 55 (20); Exact mass calcd for C_17_H_21_NO_2_S *m/z* 303.1293; EI-HRMS *m/z* 303.1295.

*Trans-6-ethyl-2-(phenylthio)-1,6,7,8,9,9a-hexahydro-4-quinolizinone* (**6**). To a solution of compound **5** (130 mg, 0.43 mmol) in 1,2-dichloroethane (2.5 mL) under nitrogen was added dropwise PBr_3_ (0.24 mL, 2.57 mmol) at reflux for 3 h. The mixture was cooled in an ice bath, and saturated NaHCO_3_ solution was then slowly added. The liquid layer was extracted three times with ethyl acetate. The organic solution was concentrated under reduced pressure and dried (MgSO_4_) to give the crude product, to which were added AIBN (28 mg, 0.17 mmol), degassed toluene (2.5 mL) and Bu_3_SnH (0.13 mL, 0.47 mmol). The mixture was then heated at reflux for 2 h, and the solvent was removed under reduced pressure. The residue was purified by flash chromatography on silica gel prewashed with Et_3_N using ethyl acetate/hexane (1:4) and 5% Et_3_N as eluent to give product **6** (75 mg, 61%). Colorless oil; ^1^H-NMR (CDCl_3_) δ 7.51–7.37 (5H, m), 5.35 (1H, s), 4.49–4.45 (1H, m), 3.63–3.53 (1H, m), 2.55 (1H, dd, *J* = 17.1, 6.3 Hz), 2.31 (1H, ddd, *J* = 17.1, 9.3, 1.2 Hz), 1.71–1.41 (8H, m), 0.86 (3H, t, *J* = 7.5 Hz); ^13^C-NMR (CDCl_3_) δ 164.3, 150.3, 135.2, 129.7 (×2), 128.4, 115.8, 50.4, 49.5, 35.2, 32.9, 26.0, 21.8, 18.4, 11.0; IR (neat) 3057, 2935, 2872, 1635, 1600, 1408, 1306, 1084, 856, 751, 692 cm^−1^; EI-MS (rel. intensity) *m/z* 287 (M^+^, 8), 259 (31), 258 (100); Exact mass calcd for C_17_H_21_NOS *m/z* 287.1344; EI-HRMS *m/z* 287.1345.

*Trans-6-propyl-2-(phenylthio)-1,6,7,8,9,9a-hexahydro-4-quinolizinone* (**7**). To a solution of compound **5** (112 mg, 0.37 mmol) in 1,2-dichloroethane (3 mL) under nitrogen was added dropwise PBr_3_ (0.21 mL, 2.22 mmol) at reflux for 3 h. The mixture was cooled in an ice bath, and saturated NaHCO_3_ solution was then slowly added. The liquid layer was extracted three times with ethyl acetate. The organic solution was concentrated under reduced pressure and dried (MgSO_4_) to give the crude product, to which were added THF (1 mL). To a mixture of CuI (211 mg, 1.11 mmol) in THF (1 mL) at 0 °C was added dropwise a solution of MeLi (1.5M in THF, 1.5 mL, 2.22 mmol). After stirring at 0 °C for 30 min, the mixture added to the prepared solution of crude product at −78 °C. The reaction mixture was slowly warmed to 0 °C in 2 h, and quenched with saturated ammonium chloride. The aqueous solution was extracted three times with ethyl acetate. The organic solution was removed under reduced pressure and dried (MgSO_4_). The residue was purified by flash chromatography on silica gel prewashed with Et_3_N using ethyl acetate/hexane (1:5) and 5% Et_3_N as eluent to give product **7** (63 mg, 57%). Colorless oil; ^1^H-NMR (CDCl_3_) δ 7.51–7.38 (5H, m), 5.33 (1H, d, *J* = 0.9 Hz), 4.62–4.56 (1H, m), 3.65–3.55 (1H, m), 2.57 (1H, dd, *J* = 16.8, 6.0 Hz), 2.32 (1H, ddd, *J* = 16.8, 9.0, 1.5 Hz), 1.75–1.40 (8H, m), 1.34–1.22 (2H, m), 0.90 (3H, t, *J* = 7.2 Hz); ^13^C-NMR (CDCl_3_) δ 164.3, 150.4, 135.4, 129.8 (×2), 128.6, 115.9, 49.7, 48.9, 35.3, 33.0, 31.3, 26.8, 19.8, 18.7, 14.1; IR (neat) 3052, 2938, 2870, 1637, 1598, 1411, 1319, 1266, 1086, 858, 738, 692 cm^–1^; EI-MS (rel. intensity) *m/z* 301 (M^+^, 7), 259 (30), 258 (100), 164 (22); Exact mass calcd for C_18_H_23_NOS *m/z* 301.1500; EI-HRMS *m/z* 301.1497.

*Trans-6-ethyl-1,6,7,8,9,9a-hexahydro-4-quinolizinone* (**8**). A mixture of compound **6** (140.2 mg, 0.49 mmol) and a W-2 Raney-Ni (1.1 g) in 95% EtOH (5 mL) was heated at reflux under nitrogen for 2 h. The solid was filtered off, and the residue was evaporated under vacuum. The crude product was purified by flash chromatography on silica gel prewashed with Et_3_N using ethyl acetate/hexane (1:1) and 5% Et_3_N as eluent to give product **8** (69.1 mg, 78%). Yellow oil; ^1^H-NMR (CDCl_3_) δ 4.86–4.79 (1H, m), 3.40–3.31 (1H, m), 2.46–2.27 (2H, m), 2.00–1.92 (1H, m), 1.82–1.75 (1H, m), 1.72–1.40 (9H, m), 1.32–1.23 (1H, m), 0.85 (3H, t, *J* = 7.2 Hz); ^13^C-NMR (CDCl_3_) δ 169.4, 51.0, 48.9, 34.1, 33.2, 30.7, 27.2, 22.4, 18.9, 18.8, 10.6; IR (neat) 2937, 2871, 1634, 1463, 1347, 1268, 1050, 791 cm^–1^; EI-MS (rel. intensity) *m/z* 181 (M^+^, 3), 153 (29), 152 (100), 41 (30); Exact mass calcd for C_11_H_19_NO *m/z* 181.1467; EI-HRMS *m/z* 181.1474.

*Trans-6-propyl-1,6,7,8,9,9a-hexahydro-4-quinolizinone* (**9**). Using a procedure similar to that for the preparation of compound **8**, compound **7** (39.1 mg, 0.13 mmol) gave product **9** (20.3 mg, 80%). Yellow oil; ^1^H-NMR (CDCl_3_) δ 4.94–4.87 (1H, m), 3.42–3.33 (1H, m), 2.45–2.26 (2H, m), 1.97–1.91 (1H, m), 1.82–1.38 (10H, m), 1.32–1.18 (3H, m), 0.92 (3H, t, *J* = 7.2 Hz); ^13^C-NMR (CDCl_3_) δ 169.4, 51.1, 47.5, 34.3, 33.3, 32.0, 30.8, 27.8, 19.5, 19.1, 18.9, 14.2; IR (neat) 3054, 2944, 2872, 1621, 1450, 1265, 1092, 705 cm^–1^; EI-MS (rel. intensity) *m/z* 195 (M^+^, 7), 153 (14), 152 (100); Exact mass calcd for C_12_H_21_NO *m/z* 195.1623; EI-HRMS *m/z* 195.1620.

*(4S/9aS,4R/9aR)-4-Ethyloctahedro-1H-quinolizine* (**10**). To a solution of compound **8** (17.1 mg, 0.09 mmol) in THF (1 mL) under nitrogen was added dropwise LiAlH_4_ (2.4 M in THF, 0.16 mL, 0.38 mmol) at 0 °C. After stirring at 0 °C for 3 h, the solvent was removed under reduced pressure. Saturated NaHCO_3_ solution was then slowly added. The mixture was extracted three times with ethyl acetate. The organic solution was carefully concentrated under reduced pressure and dried (MgSO_4_) to give the crude product, which was purified by flash chromatography on silica gel prewashed with Et_3_N using ethyl acetate/hexane (1:10) and 5% Et_3_N as eluent to give product **10** (14.2 mg, 90%). Yellow liquid; ^1^H-NMR (CDCl_3_) δ 2.67–2.48 (2H, m), 2.30–2.26 (1H, m), 1.73–1.14 (15H, m), 0.84 (3H, t, *J* = 7.5 Hz); ^13^C-NMR (CDCl_3_) δ 61.5, 54.1, 52.1, 34.3, 34.0, 27.7, 26.2, 24.7, 18.7, 15.0, 12.0; IR (neat) 2931, 2865, 2800, 1445, 1096 cm^–1^; ESI-MS (rel. intensity) *m/z* 168 (M^+^+H, 100); Exact mass calcd for C_11_H_21_N *m/z* 167.1674; ESI-HRMS *m/z* 167.1664.

*(4S/9aS,4R/9aR)-4-Propyloctahedro-1H-quinolizine* (**11**). Using a procedure similar to that for the preparation of compound **10**, compound **9** (26.3 mg, 0.13 mmol) gave product **11** (22.2 mg, 92%). Yellow liquid; ^1^H-NMR (CDCl_3_) δ 2.77–2.71 (1H, m), 2.62–2.47 (2H, m), 2.33–2.26 (1H, m), 1.70–1.11 (16H, m), 0.92 (3H, t, *J* = 7.2 Hz); ^13^C-NMR (CDCl_3_) δ 59.7, 54.1, 52.0, 34.3, 34.0, 28.5, 26.2, 24.8, 24.7, 21.0, 18.8, 14.5; IR (neat) 2933, 2866, 2808, 1449, 1096 cm^–1^; FAB-MS (rel. intensity) *m/z* 182 (M^+^+H, 14), 127 (27), 92 (32), 82 (55), 73 (51), 71 (62), 63 (87), 54 (69), 53 (100); Exact mass calcd for C_12_H_23_N *m/z* 181.1830; FAB-HRMS *m/z* 181.1833.

*(4R/6S/9aR,4S/6R/9aS)-4-Methyl-2-(phenylthio)-6-propyl-1,6,7,8,9,9a-hexahydroquinolizine* (**12**), *(4S/6S/9aR,4R/6R/9aS)-4-methyl-2-(phenylthio)-6-propyl-1,6,7,8,9,9a-hexahydroquinolizine* (**13**) *and trans-2-Methyl-6-propyl-1,6,7,8,9,9a-hexahydro-4-quinolizinone* (**14**). To a stirred solution of compound **7** (108.1 mg, 0.36 mmol) in THF (9.5 mL) was added MeMgBr (3M in THF, 0.48 mL, 1.43 mmol) dropwise at room temperature under nitrogen, followed by heating at 65 °C for 2.5 h. The reaction was then quenched with AcOH (0.1 mL) at 0 °C with stirring for about 10 min, then cooled to −50 °C. NaBH_4_ (78 mg, 2.06 mmol) and MeOH (2 mL) were then added sequentially with stirring at −50 °C for 2.5 h. The mixture was slowly warmed to 0 °C in 2 h. The solvent was removed under reduced pressure. Saturated NaHCO_3_ solution was then added. The mixture was extracted three times with CH_2_C_l2_. The organic solution was carefully concentrated under reduced pressure and dried (MgSO_4_) to give the crude product, which was purified by flash chromatography on silica gel prewashed with hexane using ethyl acetate/hexane (1:10) and 5% Et_3_N as eluent to give compound **12** (37.2 mg, 34%), compound **13** (8.4 mg, 8%) and compound **14** (28.2 mg, 38%). Compound **12**: yellow liquid; ^1^H-NMR (CDCl_3_) δ 7.35–7.27 (4H, m), 7.24–7.21 (1H, m), 5.97–5.95 (1H, m), 3.65–3.62 (1H, m), 3.36–3.29 (1H, m), 2.74–2.66 (1H, m), 2.25 (1H, dd, *J* = 17.4, 7.8 Hz), 1.96 (1H, dd, *J* = 17.4, 5.1 Hz), 1.74–1.22 (10H, m), 1.19 (3H, d, *J* = 6.6 Hz), 0.90 (3H, t, *J* = 7.2 Hz); ^13^C-NMR (CDCl_3_) δ 135.1, 134.7, 130.5, 129.0, 128.2, 126.6, 53.0, 50.5, 48.6, 35.6, 31.0, 27.9, 27.7, 21.2, 20.1, 19.2, 14.6; IR (neat) 2954, 2930, 2867, 1439, 1378, 1161, 1067, 1024, 744, 692 cm^–1^; ESI-MS (rel. intensity) *m/z* 302 (M^+^+H, 50), 300 (100), 298 (12); Exact mass calcd for C_19_H_27_NS *m/z* 301.1864; ESI-HRMS *m/z* 301.1872. Compound **13**: yellow liquid; ^1^H-NMR (CDCl_3_) δ 7.33–7.27 (4H, m), 7.23–7.18 (1H, m), 5.83 (1H, d, *J* = 1.2 Hz), 3.47–3.42 (1H, m), 3.14–3.11 (1H, m), 2.76–2.66 (1H, m), 2.06–2.00 (2H, m), 1.79–1.21 (10H, m), 1.17 (3H, d, *J* = 6.6 Hz), 0.94 (3H, t, *J* = 7.2 Hz); ^13^C-NMR (CDCl_3_) δ 135.8, 134.6, 130.6, 129.0, 127.5, 126.6, 53.3, 52.2, 50.7, 38.8, 34.4, 28.6, 24.2, 21.0, 20.0, 18.2, 14.5; IR (neat) 2955, 2929, 2867, 1439, 1371, 1133, 1068, 1025, 740, 691 cm^–1^; ESI-MS (rel. intensity) *m/z* 302 (M^+^+H, 96), 300 (100), 298 (11); Exact mass calcd for C_19_H_27_NS *m/z* 301.1864; ESI-HRMS *m/z* 301.1881. Compound **14**: yellow oil; ^1^H-NMR (CDCl_3_) δ 5.69 (1H, q, *J* = 1.2 Hz), 4.68–4.62 (1H, m), 3.63–3.53 (1H, m), 2.39 (1H, dd, *J* = 17.7, 6.9 Hz), 2.08 (1H, dd, *J* = 17.7, 8.4 Hz), 1.85 (3H, s), 1.70–1.23 (10H, m), 0.92 (3H, t, *J* = 7.2 Hz); ^13^C-NMR (CDCl_3_) δ 165.4, 147.7, 120.4, 49.1, 48.6, 36.0, 33.5, 31.2, 26.8, 22.6, 19.7, 18.8, 14.1; IR (neat) 2934, 2869, 1677, 1424, 1327, 1040 cm^–1^; EI-MS (rel. intensity) *m/z* 207 (M^+^, 7), 165 (17), 164 (100), 82 (6); Exact mass calcd for C_13_H_21_NO *m/z* 207.1623; EI-HRMS *m/z* 207.1623.

*(4R/6S/9aS,4S/6R/9aR)-4-Methyl-6-propyl-2,3,4,6,7,8,9,9a-octahydro-1H-quinolizine* (**15**). A mixture of compound **12** (23.4 mg, 0.08 mmol) and a W-2 Raney-Ni (175 mg) in 95% EtOH (3 mL) was heated at reflux under nitrogen for 2 h. The solid was filtered off, and the residue was evaporated under vacuum. The crude product was purified by flash chromatography on silica gel prewashed with hexane using hexane and 5% Et_3_N as eluent to give product **15** (11.4 mg, 75%). Yellow liquid; ^1^H-NMR (CDCl_3_) δ 3.22–3.18 (1H, m), 3.14–3.00 (2H, m), 1.90–1.81 (2H, m), 1.70–1.54 (9H, m), 1.34–1.24 (4H, m), 1.16–1.12 (1H, m), 1.06 (3H, d, *J* = 6.0 Hz), 0.91 (3H, t, *J* = 7.2 Hz); ^13^C-NMR (CDCl_3_) δ 52.4, 49.4, 47.1, 34.4, 33.7, 30.2, 24.6, 22.5, 21.0, 20.3 (×2), 20.0, 14.5; IR (neat) 2931, 2864, 2795, 1457, 1365 cm^–1^; ESI-MS (rel. intensity) *m/z* 196 (M^+^+H, 100); Exact mass calcd for C_13_H_25_N *m/z* 195.1987; ESI-HRMS *m/z* 195.1968.

*(4S/6S/9aS,4R/6R/9aR)-4-Methyl-6-propyl-2,3,4,6,7,8,9,9a-octahydro-1H-quinolizine* (**16**). A mixture of compound **13** (34.4 mg, 0.11 mmol) and a W-2 Raney-Ni (258 mg) in 95% EtOH (5 mL) was heated at reflux under nitrogen for 2 h. The solid was filtered off, and the residue was evaporated under vacuum. The crude product was purified by flash chromatography on silica gel prewashed with hexane using hexane and 5% Et_3_N as eluent to give product **16** (16.3 mg, 73%) as a yellow liquid. The spectral data of compound **16** were identical with the literature data [37].

*Trans-6-propyl-2-(phenylsulfonyl)-1,6,7,8,9,9a-hexahydro-4-quinolizinone* (**17**). To a solution of compound **7** (69.5 mg, 0.23 mmol) in CH_2_Cl_2_ (2 mL) at 0 °C was added dropwise another solution of mCPBA (60% in H_2_O, 264 mg, 0.92 mmol) in CH_2_Cl_2_ (3 mL). The reaction mixture was slowly warmed to room temperature and stirred for another 4 h, then diluted with more CH_2_Cl_2_. The solution was washed with saturated aqueous sodium thiosulfate and saturated sodium bicarbonate solution. The organic solution was removed under reduced pressure and dried (MgSO_4_). The residue was purified by flash chromatography on silica gel prewashed with Et_3_N using ethyl acetate/hexane (1:3) and 5% Et_3_N as eluent to give product **17** (60.4 mg, 79%). Colorless oil; ^1^H-NMR (CDCl_3_) δ 7.90 (2H, d, *J* = 7.2 Hz), 7.73–7.68 (1H, m), 7.60 (2H, t, *J* = 7.2 Hz), 6.62 (1H, s), 4.65–4.59 (1H, m), 3.71–3.61 (1H, m), 2.73 (1H, dd, *J* = 17.4, 6.6 Hz), 2.26 (1H, ddd, *J* = 17.4, 8.7, 1.8 Hz), 1.70–1.43 (7H, m), 1.38–1.20 (3H, m), 0.90 (3H, t, *J* = 7.2 Hz); ^13^C-NMR (CDCl_3_) δ 162.1, 147.2, 137.3, 134.4, 129.6, 128.5, 127.8, 49.7, 49.6, 33.0, 31.3, 28.3, 26.6, 19.6, 18.3, 13.9; IR (neat) 3064, 2936, 2870, 1665, 1623, 1428, 1322, 1155, 1081, 858, 759, 689 cm^−1^; FAB-MS (rel. intensity) *m/z* 334 (M^+^+H, 100), 332 (14), 290 (17), 126 (16), 105 (20); Exact mass calcd for C_18_H_23_NO_3_S *m/z* 333.1399; FAB-HRMS *m/z* 333.1400.

*Trans-6-(2-hydroxyethyl)-1,6,7,8,9,9a-hexahydro-4-quinolizinone* (**18**). Using a procedure similar to that for the preparation of compound **8**, compound **5** (309 mg, 1.02 mmol) gave product **18** (157 mg, 78%). Yellow oil; ^1^H-NMR (CDCl_3_) δ 5.00–4.96 (1H, m), 3.86 (1H, OH), 3.64–3.57 (1H, m), 3.32–3.23 (2H, m), 2.51–2.32 (2H, m), 2.05–1.46 (11H, m), 1.38–1.26 (1H, m); ^13^C-NMR (CDCl_3_) δ 171.4, 58.0, 51.7, 44.4, 34.0, 33.1, 32.5, 30.8, 29.2, 19.2, 19.0; IR (neat) 3400, 2938, 2867, 1610, 1449, 1064 cm^–1^; EI-MS (rel. intensity) *m/z* 197 (M^+^, 10), 153 (26), 152 (100); Exact mass calcd for C_11_H_19_NO_2_ *m/z* 197.1416; EI-HRMS *m/z* 197.1416.

*(2R/4S/9aS,2S/4R/9aR)- and (2S/4S/9aS,2R/4R9aR)-2-Methyl-6-propyl-1,2,3,6,7,8,9,9a-octa-hydro-4-quinolizinone* (**19**). A mixture of compound **14** (22.3 mg, 0.11 mmol) and PtO_2_ (4 mg) in EtOAc (3 mL) was stirred vigorously under a balloon of hydrogen at room temperature for 12 h. The reaction mixture was then filtered with Celite, washed with ethyl acetate, dried (MgSO_4_), and evaporated under vacuum to give product **19** (20.6 mg, 92%). Yellow liquid; ^1^H-NMR (CDCl_3_) δ 4.94–4.84 (m), 3.53–3.45 (m), 3.40–3.31 (m), 2.50–2.42 (m), 2.06–1.87 (m), 1.81–1.03 (m), 0.99–0.88 (m); ^13^C-NMR (CDCl_3_,) δ 169.5, 168.9, 51.4, 49.0, 48.0, 47.5, 41.6, 40.8, 39.9, 37.1, 34.9, 33.8, 32.2, 31.9, 27.9, 27.8, 26.3, 23.9, 21.2, 20.3, 19.8, 19.7, 19.6, 18.8, 14.2 (×2); IR (neat) 3052, 2936, 2872, 1623, 1452, 1266, 1088, 738 cm^–1^; EI-MS (rel. intensity) *m/z* 209 (M^+^, 14), 167 (38), 166 (100); Exact mass calcd for C_13_H_23_NO *m/z* 209.1780; EI-HRMS *m/z* 209.1784.

*Trans-6-propyl-1,6,7,8,9,9a-hexahydro-4-quinolizinone* (**20**). To a solution of compound **17** (56.2 mg, 0.17 mmol) in dried THF (4 mL) was added 6% sodium amalgam (1.68 mmol) and two drops of concentrated phosphoric acid. The mixture was heated at reflux for 2 h. Upon cooling the mixture was filtered through Celite, rinsed with ethyl acetate, and evaporated under vacuum. The residue was purified by flash chromatography on silica gel prewashed with Et_3_N using ethyl acetate/hexane (1:2) and 5% Et_3_N as eluent to give product **20** (25.1 mg, 77%). Yellow oil; ^1^H-NMR (CDCl_3_) δ 6.43–6.37 (1H, m), 5.88 (1H, dt, *J* = 9.6 1.8 Hz), 4.71–4.64 (1H, m), 3.65–3.56 (1H, m), 2.51 (1H, dddd, *J* = 18.0, 7.2, 5.1, 1.8 Hz), 2.15 (1H, dddd, *J* = 18.0, 8.4, 3.6, 2.4 Hz), 1.72–1.43 (8H, m), 1.37–1.25 (2H, m), 0.93 (3H, t, *J* = 7.2 Hz); ^13^C-NMR (CDCl_3_) δ 164.6, 136.8, 125.1, 49.2, 48.8, 33.4, 31.2, 30.8, 26.8, 19.7, 18.8, 14.1; IR (neat) 2934, 2870, 1667, 1614, 1424, 1307, 1157 cm^–1^; ESI-MS (rel. intensity) *m/z* 194 (M^+^+H, 100); Exact mass calcd for C_12_H_19_NO *m/z* 193.1467; ESI-HRMS *m/z* 193.1459.

*Trans-6-[2-oxoproyl]-2-(phenylthio)-1,6,7,8,9,9a-hexahydro-4-quinolizinone* (**21**). To a stirred solution of compound **4b** (22.2 mg, 0.07 mmol) in THF (1.5 mL) was added (trimethylsilyl)methylmagnesium chloride (1.3 M in THF, 0.15 mL, 0.20 mmol) at room temperature under nitrogen for 3 h. The reaction solution was washed with 1N HCl solution. The aqueous layer was extracted three times with ethyl acetate. The organic solution was concentrated under reduced pressure and dried (MgSO_4_) to give the crude product, which was purified by flash chromatography on silica gel prewashed with hexane using ethyl acetate/hexane (1:4) and 5% Et_3_N as eluent to give compound **21** (13.4 mg, 64%). Colorless oil; ^1^H-NMR (CDCl_3_) δ 7.50–7.38 (5H, m), 5.28 (1H, d, *J* = 1.8 Hz), 5.02–4.96 (1H, m), 3.62–3.52 (1H, m), 2.60 (2H, d, *J* = 7.2 Hz), 2.50 (1H, dd, *J* = 17.1, 6.0 Hz), 2.38 (1H, ddd, *J* = 17.1, 10.2, 1.8 Hz), 2.18 (3H, s), 1.80–1.76 (1H, m), 1.71–1.55 (4H, m), 1.47–1.38 (1H, m); ^13^C-NMR (CDCl_3_) δ 207.4, 164.7, 152.0, 135.3, 129.9, 129.8, 128.2, 115.2, 50.3, 45.7, 43.8, 35.6, 32.7, 29.8, 27.3, 18.2; IR (neat) 3057, 2938, 2864, 1712, 1639, 1599, 1408, 1303, 1168, 1023, 856, 752 cm^–1^; EI-MS (rel. intensity) *m/z* 315 (M^+^, 24), 314 (17), 272 (39), 258 (67), 56 (100); Exact mass calcd for C_18_H_21_NO_2_S *m/z* 315.1293; EI-HRMS *m/z* 315.1291.

## 4. Conclusions

In summary, we have converted *trans*-quinolizidinone **4b** into the C-6 alkylated derivatives **6**–**11**. Treatment of compound **7** with methylmagnesium bromide, followed by acidification with acetic acid and reduction with NaBH_4_ at low temperature gave selectively the C-6,9a *trans*-compound **12**, which was reacted with Raney nickel in refluxing 95% EtOH to achieve the synthesis of compound **15** which was the proposed structure of the natural product (±)-quinolizidine 195C. However, the spectral data of compound **15** differed significantly from that reported in the literature, so the true structure of (±)-quinolizidine 195C remains uncertain. We have also prepared some other functionalized quinolizidines **17**–**21** for further biological studies.

## Supplementary Material

^1^H- and ^13^C-NMR spectra of compounds **5**–**21** can be found in the online Supplementary Data.

Supplementary materials can be accessed at: http://www.mdpi.com/1420-3049/18/7/8243/s1.
