# Supplementary material for: Studies toward the First Stereoselective Total Synthesis of (±)-Quinolizidine 195C and Other Transformations
Source: Molecules. 2013 Jul 12;18(7):8243–56. doi: 10.3390/molecules18078243 (PMC6269906; doi:10.3390/molecules18078243)
Supplement: Supplementary file 1 [file molecules-18-08243-s001.pdf]

Supplementary Materials

^{1}H- and ^{13}C-NMR spectra of compounds **5–21**.

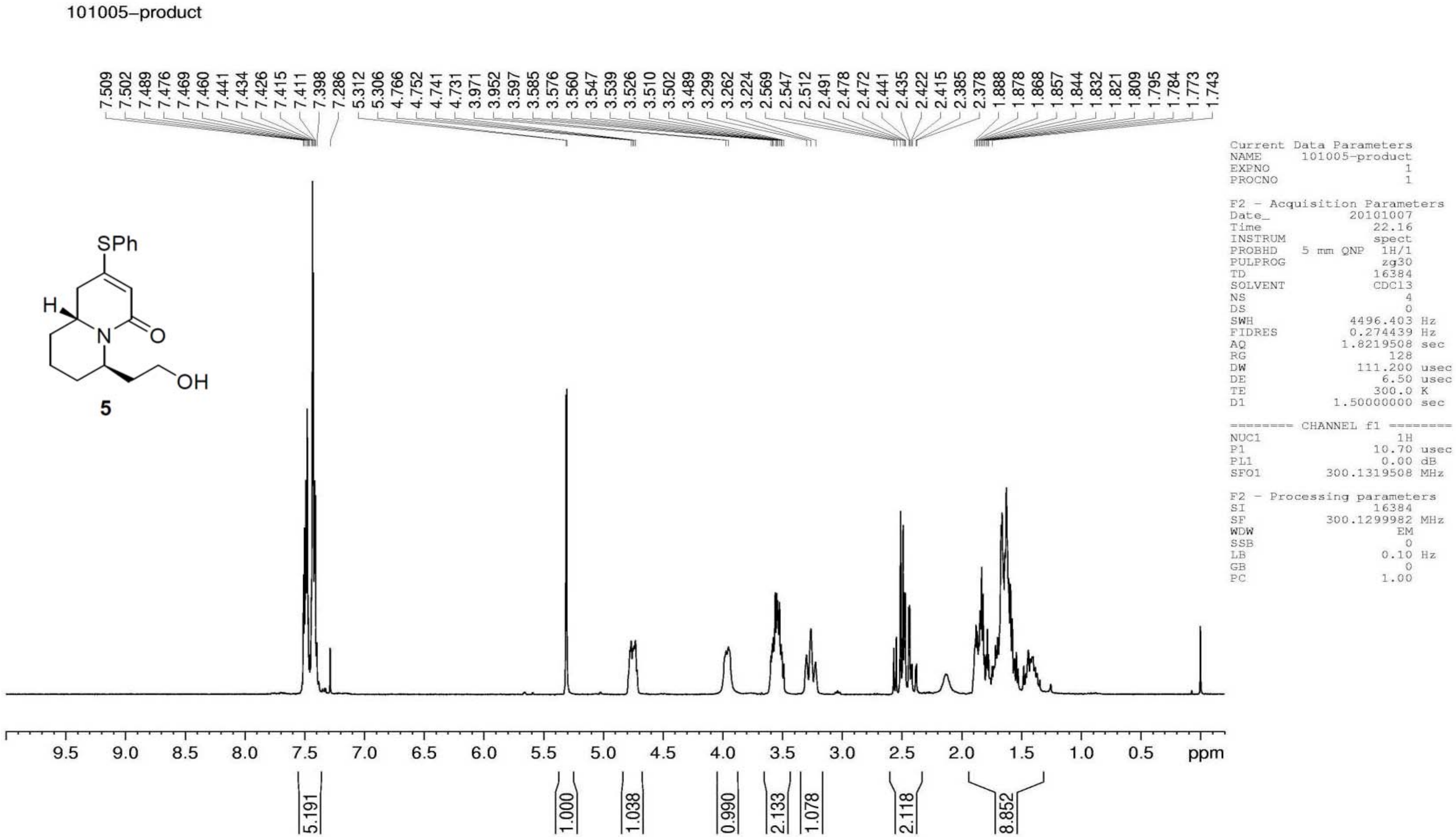

100811-product-C13

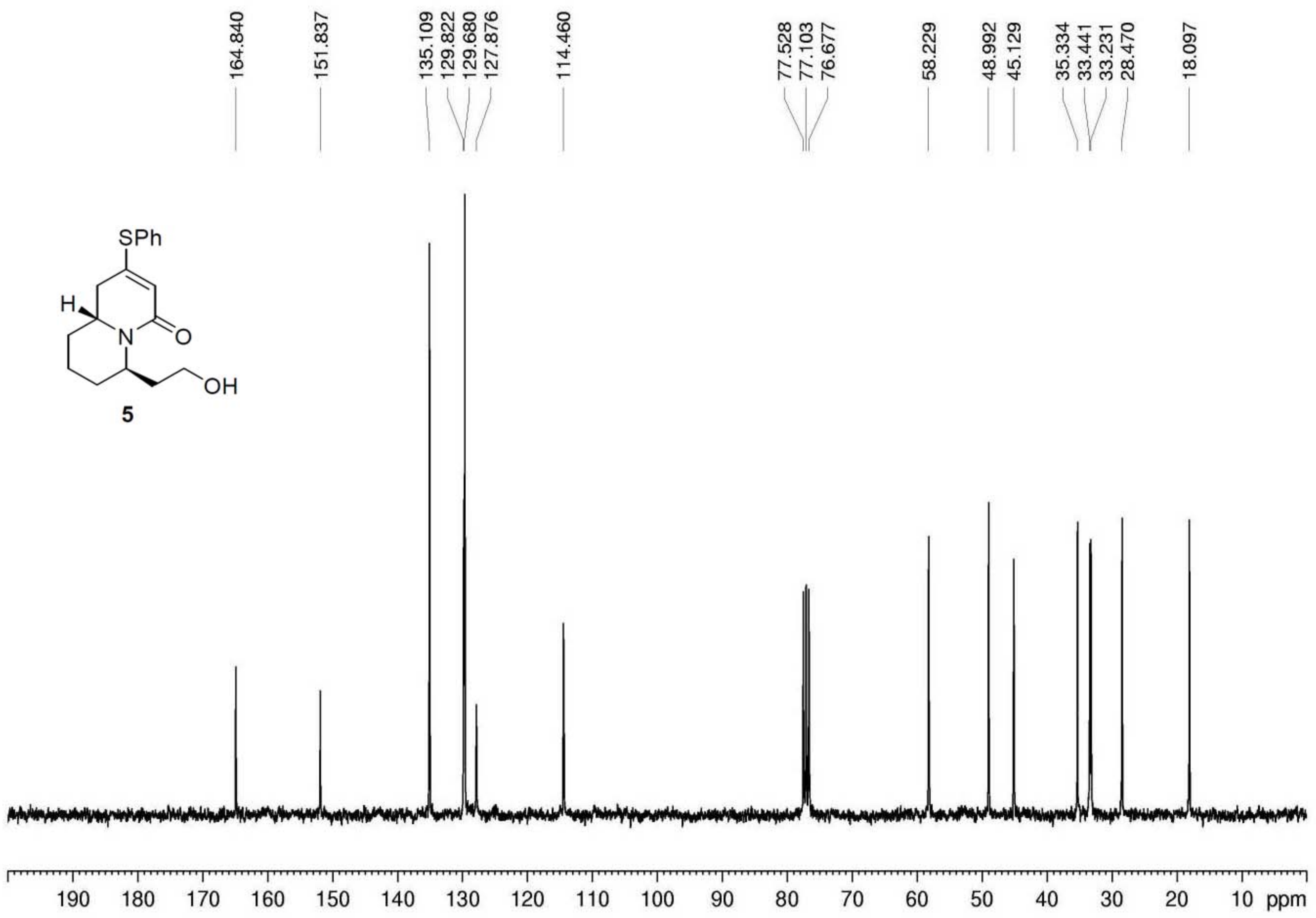

5

```
Current Data Parameters
NAME       100811-product
EXPNO                   2
PROCNO                  1

F2 - Acquisition Parameters
Date_            20100818
Time                 0.16
INSTRUM             spect
PROBHD   5 mm QNP  1H/1
PULPROG           zgpg30
TD                 65536
SOLVENT            CDCl3
NS                   128
DS                     0
SWH            18832.393 Hz
FIDRES          0.287360 Hz
AQ             1.7400308 sec
RG               14596.5
DW                26.550 usec
DE                  6.50 usec
TE                 300.0 K
D1            1.20000005 sec
d11           0.03000000 sec
d12           0.00002000 sec

======== CHANNEL f1 ========
NUC1                 13C
P1                 10.10 usec
PL1                 0.00 dB
SFO1          75.4763978 MHz

======== CHANNEL f2 ========
CPDPRG2          waltz16
NUC2                  1H
PCPD2              90.00 usec
PL2                 0.00 dB
PL12               18.10 dB
PL13               21.10 dB
SFO2         300.1313506 MHz

F2 - Processing parameters
SI                 32768
SF            75.4677591 MHz
WDW                   EM
SSB                    0
LB                  3.00 Hz
GB                     0
PC                  1.00
```

100824–product–NOE

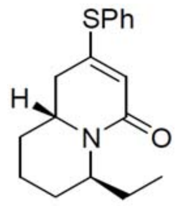

6

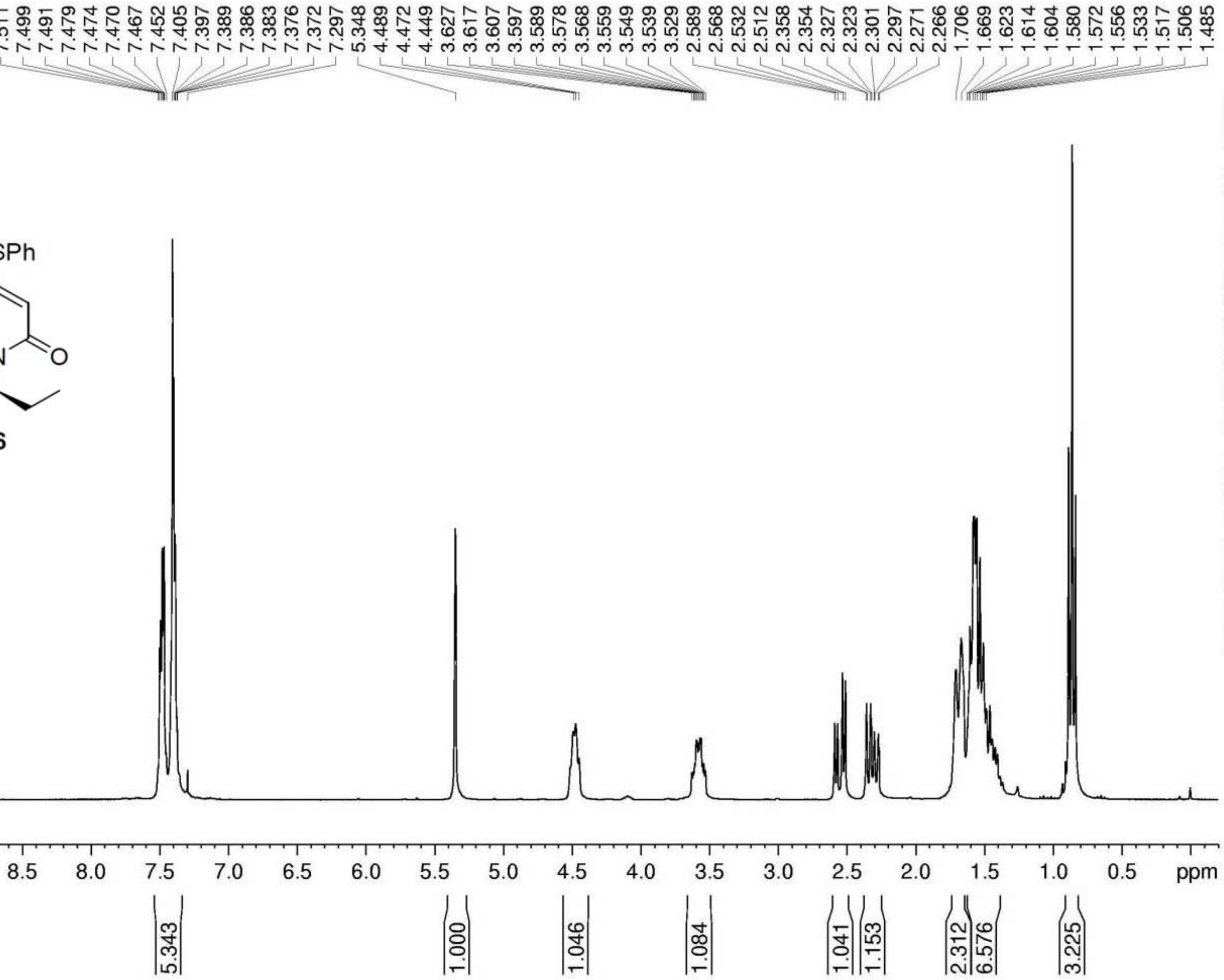


```
Current Data Parameters
NAME       100824-product-NOE
EXPNO                       5
PROCNO                      1

F2 - Acquisition Parameters
Date_                20100901
Time                    16.22
INSTRUM                 spect
PROBHD    5 mm QNP  1H/1
PULPROG                  zg30
TD                      16384
SOLVENT                 CDCl3
NS                         64
DS                          0
SWH                  4496.403 Hz
FIDRES               0.274439 Hz
AQ                  1.8219508 sec
RG                        128
DW                    111.200 usec
DE                       6.50 usec
TE                      300.0 K
D1                 1.50000000 sec

======== CHANNEL f1 ========
NUC1                       1H
P1                      10.70 usec
PL1                      0.00 dB
SFO1              300.1319508 MHz

F2 - Processing parameters
SI                      16384
SF                300.1299949 MHz
WDW                        EM
SSB                         0
LB                       0.10 Hz
GB                          0
PC                       1.00
```

100824-product-NOE-C13

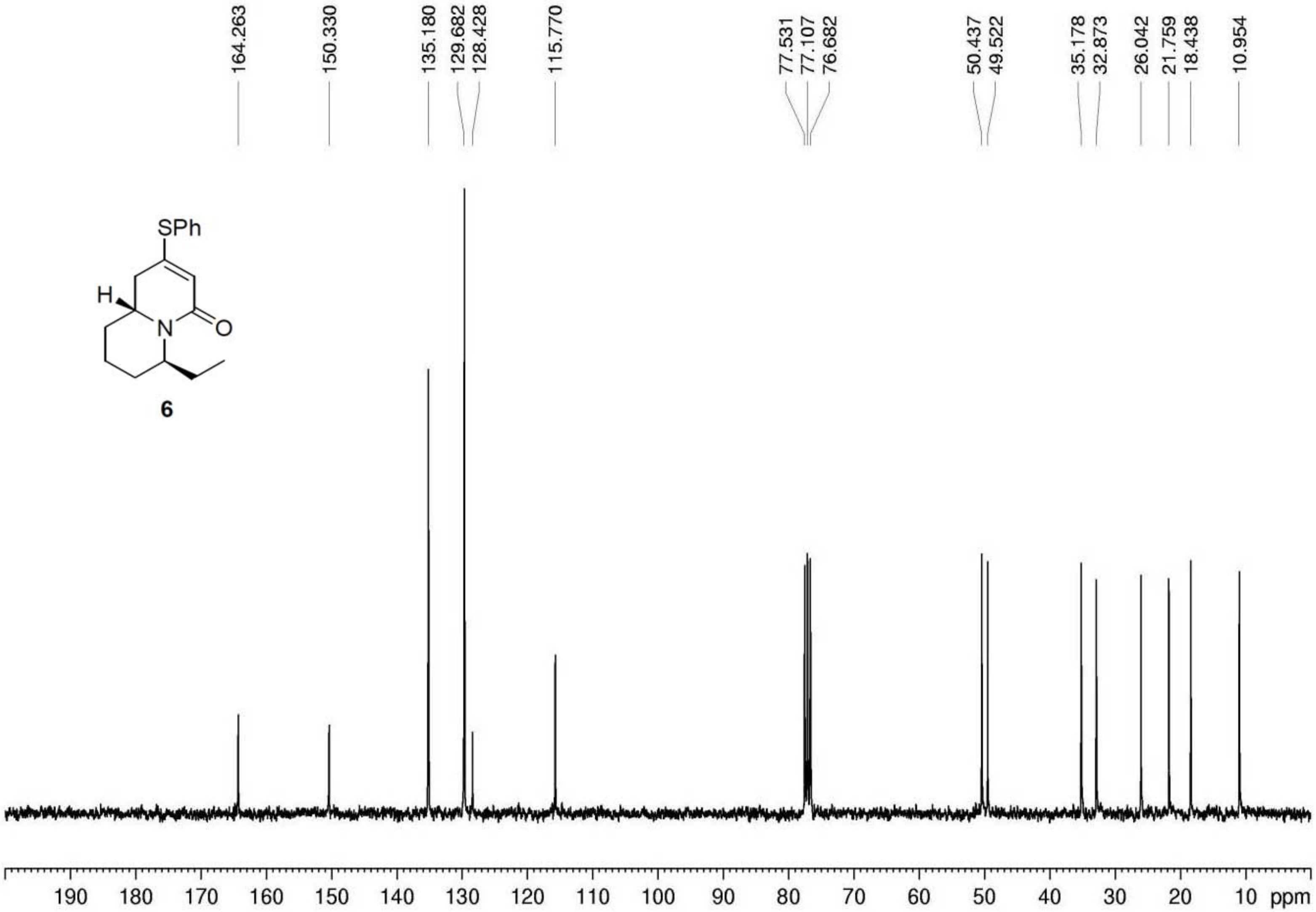


```
Current Data Parameters
NAME       100824-product-NOE
EXPNO                   2
PROCNO                  1

F2 - Acquisition Parameters
Date_            20100901
Time                16.11
INSTRUM             spect
PROBHD     5 mm QNP  1H/1
PULPROG            zgpg30
TD                  65536
SOLVENT             CDCl3
NS                    128
DS                      0
SWH             18832.393 Hz
FIDRES           0.287360 Hz
AQ              1.7400308 sec
RG                14596.5
DW                 26.550 usec
DE                   6.50 usec
TE                  300.0 K
D1             1.20000005 sec
d11            0.03000000 sec
d12            0.00002000 sec

======== CHANNEL f1 ========
NUC1                  13C
P1                  10.10 usec
PL1                  0.00 dB
SFO1           75.4763978 MHz

======== CHANNEL f2 ========
CPDPRG2           waltz16
NUC2                   1H
PCPD2               90.00 usec
PL2                  0.00 dB
PL12                18.10 dB
PL13                21.10 dB
SFO2          300.1313506 MHz

F2 - Processing parameters
SI                  32768
SF             75.4677522 MHz
WDW                    EM
SSB                     0
LB                   3.00 Hz
GB                      0
PC                   1.00
```

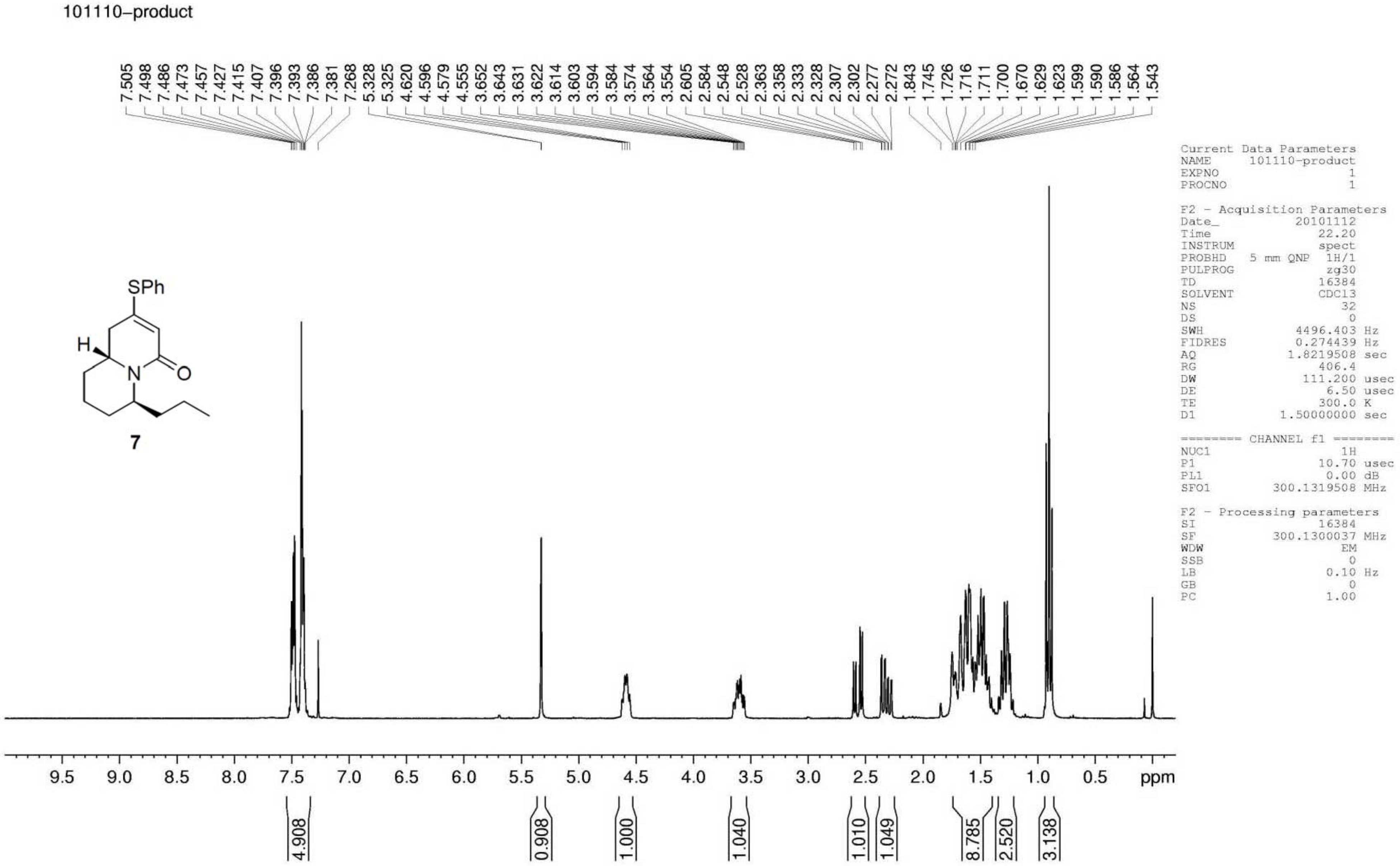

101110–product
7.505
7.498
7.486
7.473
7.457
7.427
7.415
7.407
7.396
7.393
7.386
7.381
7.268
5.328
5.325
4.620
4.596
4.579
4.555
3.652
3.643
3.631
3.622
3.614
3.603
3.594
3.584
3.574
3.564
3.554
2.605
2.584
2.548
2.528
2.363
2.358
2.333
2.328
2.307
2.302
2.277
2.272
1.843
1.745
1.726
1.716
1.711
1.700
1.670
1.629
1.623
1.599
1.590
1.586
1.564
1.543
SPh
H
N
O
7
Current Data Parameters
NAME 101110-product
EXPNO 1
PROCNO 1
F2 - Acquisition Parameters
Date_ 20101112
Time 22.20
INSTRUM spect
PROBHD 5 mm QNP 1H/1
PULPROG zg30
TD 16384
SOLVENT CDCl3
NS 32
DS 0
SWH 4496.403 Hz
FIDRES 0.274439 Hz
AQ 1.8219508 sec
RG 406.4
DW 111.200 usec
DE 6.50 usec
TE 300.0 K
D1 1.50000000 sec
======== CHANNEL f1 ========
NUC1 1H
P1 10.70 usec
PL1 0.00 dB
SFO1 300.1319508 MHz
F2 - Processing parameters
SI 16384
SF 300.1300037 MHz
WDW EM
SSB 0
LB 0.10 Hz
GB 0
PC 1.00
9.5 9.0 8.5 8.0 7.5 7.0 6.5 6.0 5.5 5.0 4.5 4.0 3.5 3.0 2.5 2.0 1.5 1.0 0.5 ppm
4.908
0.908
1.000
1.040
1.010
1.049
8.785
2.520
3.138

101110-product-C13

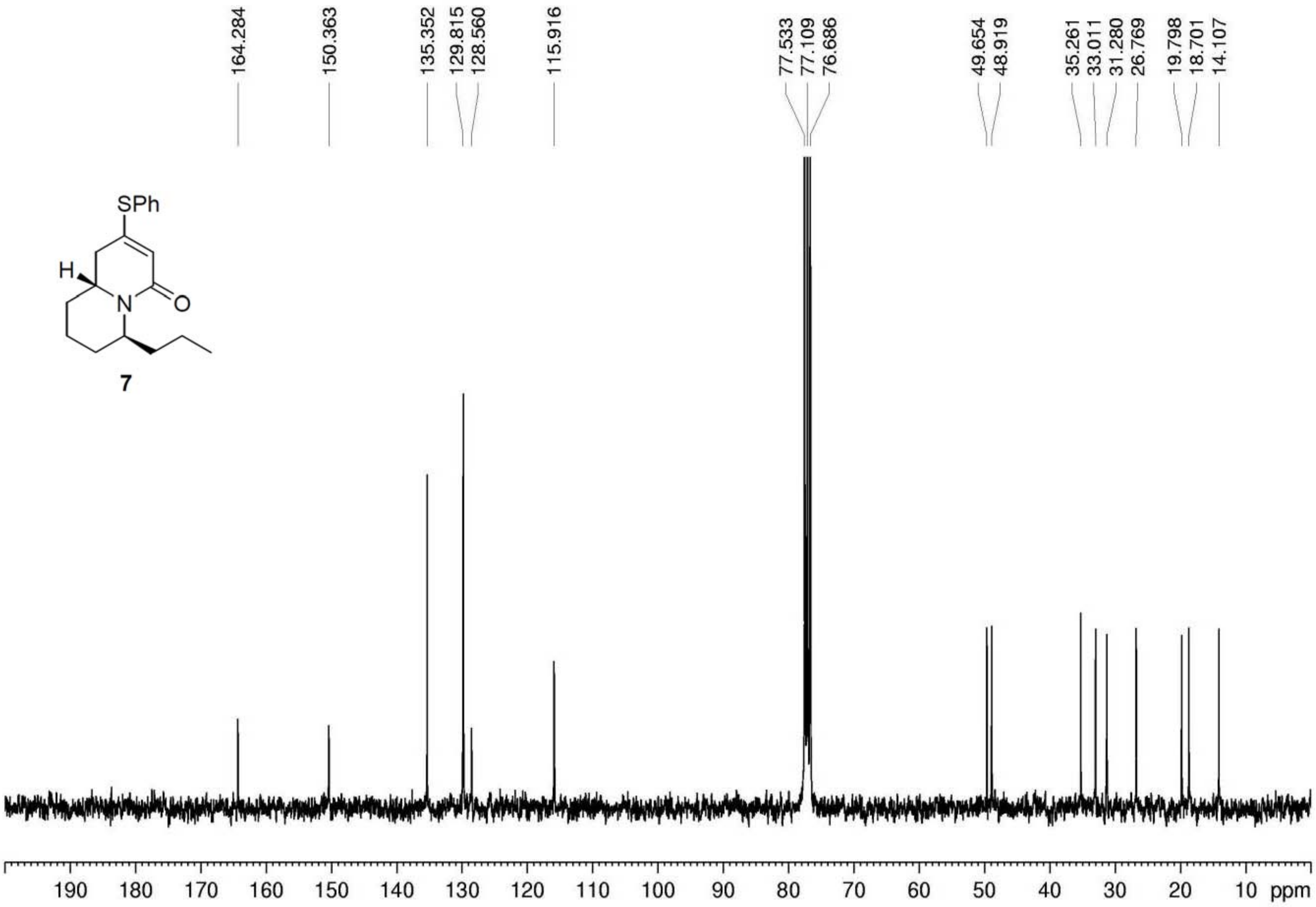


```
Current Data Parameters
NAME        101110-product
EXPNO                    2
PROCNO                   1

F2 - Acquisition Parameters
Date_             20101112
Time                 22.46
INSTRUM              spect
PROBHD    5 mm QNP  1H/1
PULPROG            zgpg30
TD                   65536
SOLVENT              CDCl3
NS                     512
DS                       0
SWH             18832.393 Hz
FIDRES           0.287360 Hz
AQ              1.7400308 sec
RG                 11585.2
DW                  26.550 usec
DE                    6.50 usec
TE                   300.0 K
D1             1.20000005 sec
d11            0.03000000 sec
d12            0.00002000 sec

======== CHANNEL f1 ========
NUC1                   13C
P1                   10.10 usec
PL1                   0.00 dB
SFO1            75.4763978 MHz

======== CHANNEL f2 ========
CPDPRG2            waltz16
NUC2                    1H
PCPD2                90.00 usec
PL2                   0.00 dB
PL12                 18.10 dB
PL13                 21.10 dB
SFO2           300.1313506 MHz

F2 - Processing parameters
SI                   32768
SF              75.4677430 MHz
WDW                     EM
SSB                      0
LB                    3.00 Hz
GB                       0
PC                    1.00
```

111116-product

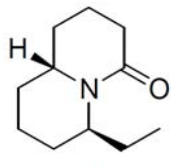

8

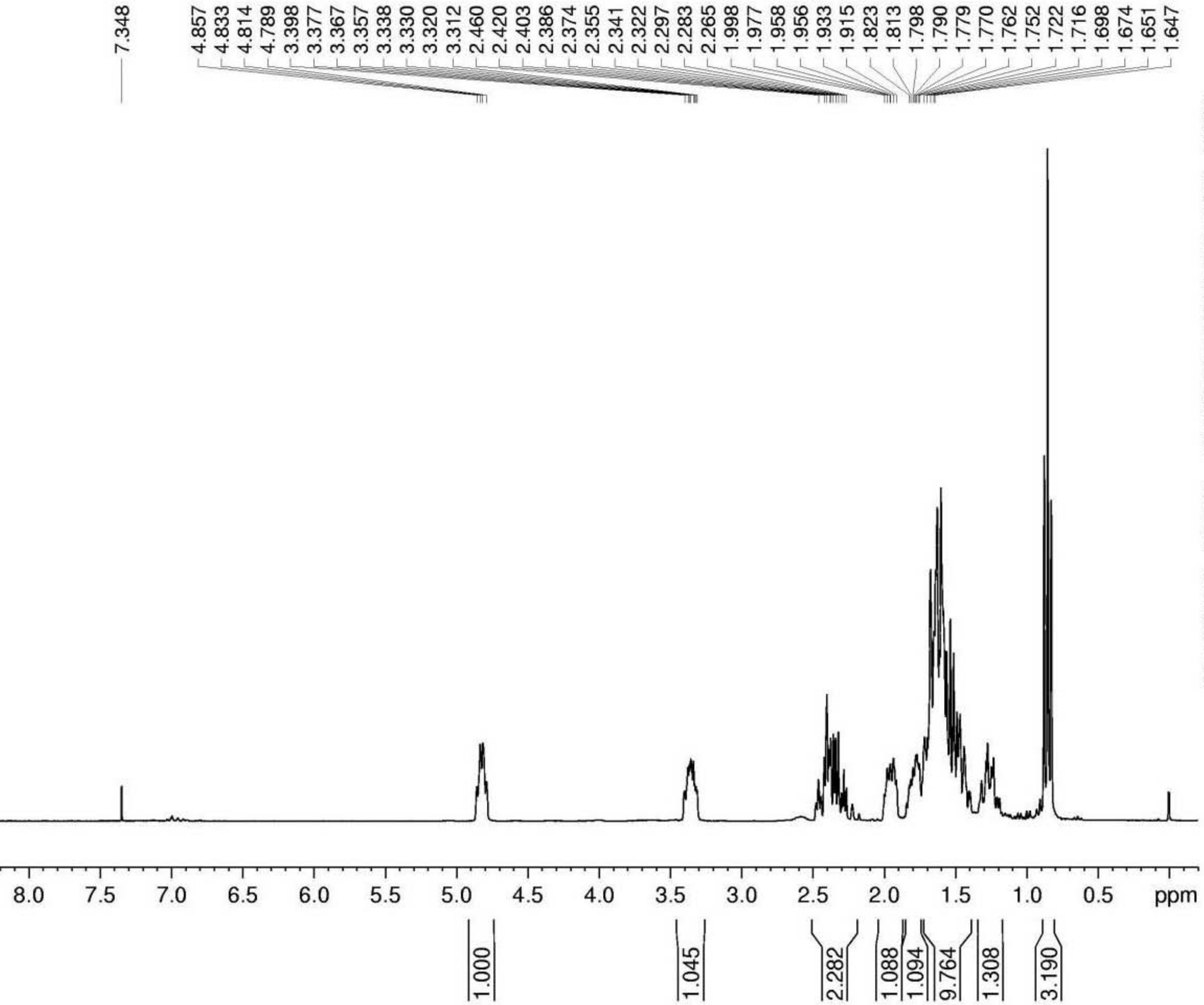


```
Current Data Parameters
NAME      111116-product
EXPNO                  1
PROCNO                 1

F2 - Acquisition Parameters
Date_           20111117
Time               21.03
INSTRUM            spect
PROBHD   5 mm QNP  1H/1
PULPROG             zg30
TD                 16384
SOLVENT            CDCl3
NS                     8
DS                     0
SWH             5411.255 Hz
FIDRES          0.330277 Hz
AQ             1.5139316 sec
RG                  71.8
DW                92.400 usec
DE                  6.50 usec
TE                 300.0 K
D1            1.50000000 sec
TD0                    1

======== CHANNEL f1 ========
NUC1                  1H
P1                 11.40 usec
PL1                 0.00 dB
SFO1         300.1319508 MHz

F2 - Processing parameters
SI                 16384
SF           300.1299794 MHz
WDW                   EM
SSB                    0
LB                  0.10 Hz
GB                     0
PC                  1.00
```

111116–product

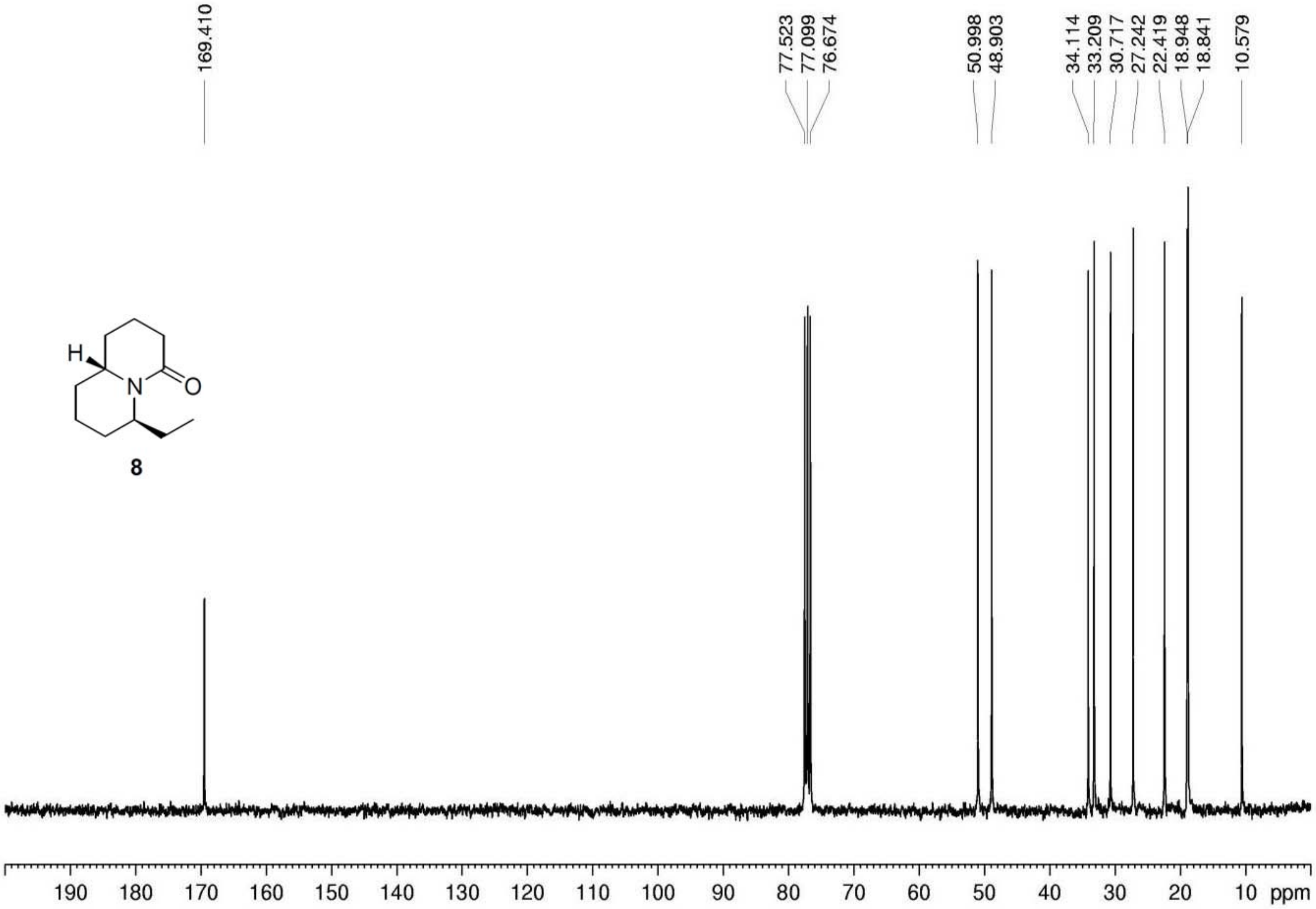


```
Current Data Parameters
NAME       111116-product
EXPNO                   2
PROCNO                  1

F2 - Acquisition Parameters
Date_            20111117
Time                21.21
INSTRUM             spect
PROBHD   5 mm QNP  1H/1
PULPROG           zgpg30
TD                 65536
SOLVENT            CDCl3
NS                   256
DS                     0
SWH            19267.822 Hz
FIDRES          0.294004 Hz
AQ             1.7007092 sec
RG               14596.5
DW                25.950 usec
DE                  6.50 usec
TE                 300.0 K
D1            2.00000000 sec
D11           0.03000000 sec
TD0                    1

======== CHANNEL f1 ========
NUC1                 13C
P1                  9.50 usec
PL1                -1.50 dB
SFO1          75.4771825 MHz

======== CHANNEL f2 ========
CPDPRG2          waltz16
NUC2                  1H
PCPD2              90.00 usec
PL2                 0.00 dB
PL12               18.00 dB
PL13               21.00 dB
SFO2         300.1313506 MHz

F2 - Processing parameters
SI                 32768
SF            75.4677524 MHz
WDW                   EM
SSB                    0
LB                  3.00 Hz
GB                     0
PC                  1.00
```

110711-2

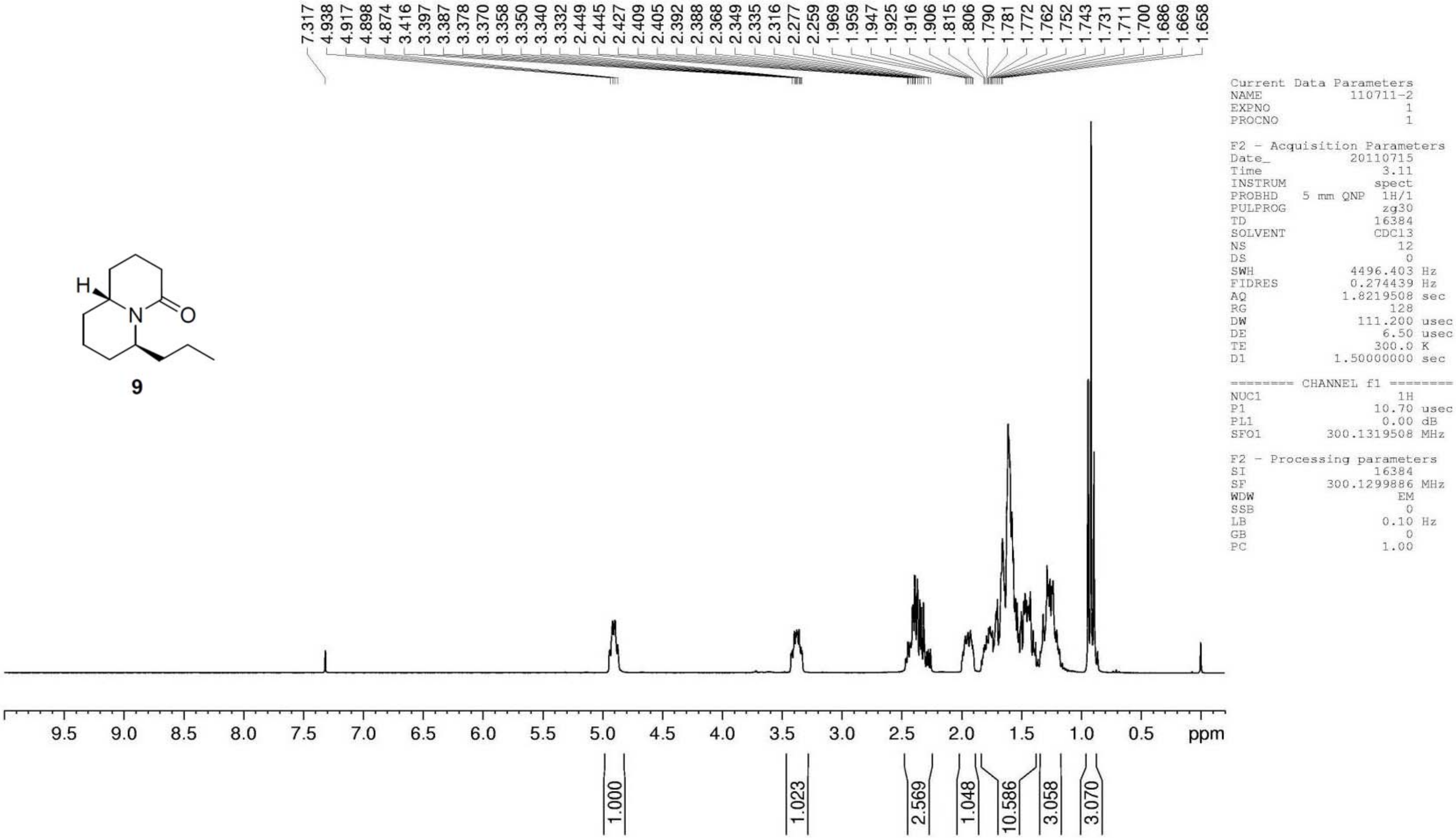
7.317
4.938
4.917
4.898
4.874
3.416
3.397
3.387
3.378
3.370
3.358
3.350
3.340
3.332
2.449
2.445
2.427
2.409
2.405
2.392
2.388
2.368
2.349
2.335
2.316
2.277
2.259
1.969
1.959
1.947
1.925
1.916
1.906
1.815
1.806
1.790
1.781
1.772
1.762
1.752
1.743
1.731
1.711
1.700
1.686
1.669
1.658
9.5 9.0 8.5 8.0 7.5 7.0 6.5 6.0 5.5 5.0 4.5 4.0 3.5 3.0 2.5 2.0 1.5 1.0 0.5 ppm
1.000
1.023
2.569
1.048
10.586
3.058
3.070
Current Data Parameters
NAME 110711-2
EXPNO 1
PROCNO 1
F2 - Acquisition Parameters
Date_ 20110715
Time 3.11
INSTRUM spect
PROBHD 5 mm QNP 1H/1
PULPROG zg30
TD 16384
SOLVENT CDCl3
NS 12
DS 0
SWH 4496.403 Hz
FIDRES 0.274439 Hz
AQ 1.8219508 sec
RG 128
DW 111.200 usec
DE 6.50 usec
TE 300.0 K
D1 1.50000000 sec
======== CHANNEL f1 ========
NUC1 1H
P1 10.70 usec
PL1 0.00 dB
SFO1 300.1319508 MHz
F2 - Processing parameters
SI 16384
SF 300.1299886 MHz
WDW EM
SSB 0
LB 0.10 Hz
GB 0
PC 1.00

9

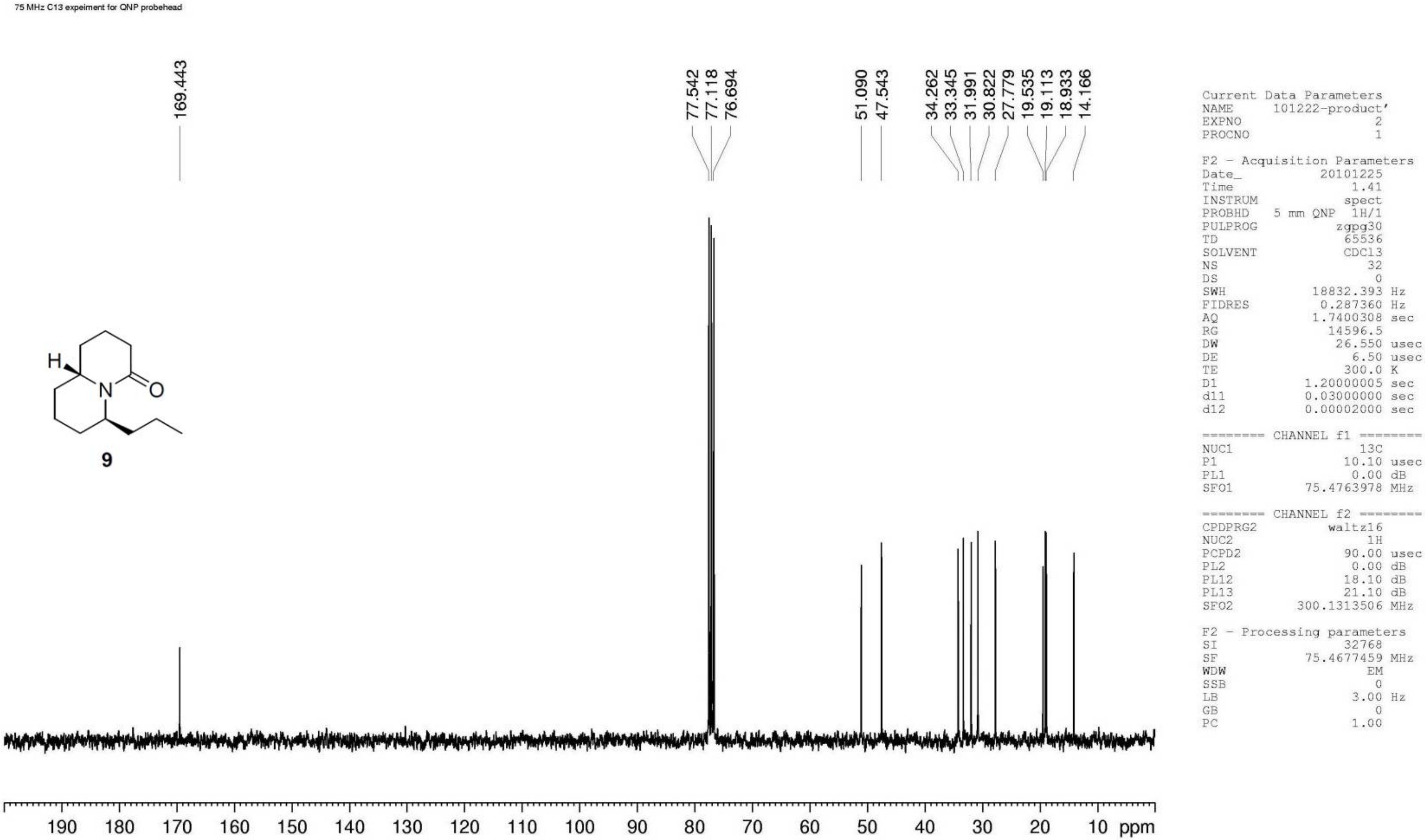
75 MHz C13 expeiment for QNP probehead
169.443
77.542
77.118
76.694
51.090
47.543
34.262
33.345
31.991
30.822
27.779
19.535
19.113
18.933
14.166
H
N
O
9
190 180 170 160 150 140 130 120 110 100 90 80 70 60 50 40 30 20 10 ppm
Current Data Parameters
NAME 101222-product'
EXPNO 2
PROCNO 1
F2 - Acquisition Parameters
Date_ 20101225
Time 1.41
INSTRUM spect
PROBHD 5 mm QNP 1H/1
PULPROG zgpg30
TD 65536
SOLVENT CDCl3
NS 32
DS 0
SWH 18832.393 Hz
FIDRES 0.287360 Hz
AQ 1.7400308 sec
RG 14596.5
DW 26.550 usec
DE 6.50 usec
TE 300.0 K
D1 1.20000005 sec
d11 0.03000000 sec
d12 0.00002000 sec
======== CHANNEL f1 ========
NUC1 13C
P1 10.10 usec
PL1 0.00 dB
SFO1 75.4763978 MHz
======== CHANNEL f2 ========
CPDPRG2 waltz16
NUC2 1H
PCPD2 90.00 usec
PL2 0.00 dB
PL12 18.10 dB
PL13 21.10 dB
SFO2 300.1313506 MHz
F2 - Processing parameters
SI 32768
SF 75.4677459 MHz
WDW EM
SSB 0
LB 3.00 Hz
GB 0
PC 1.00

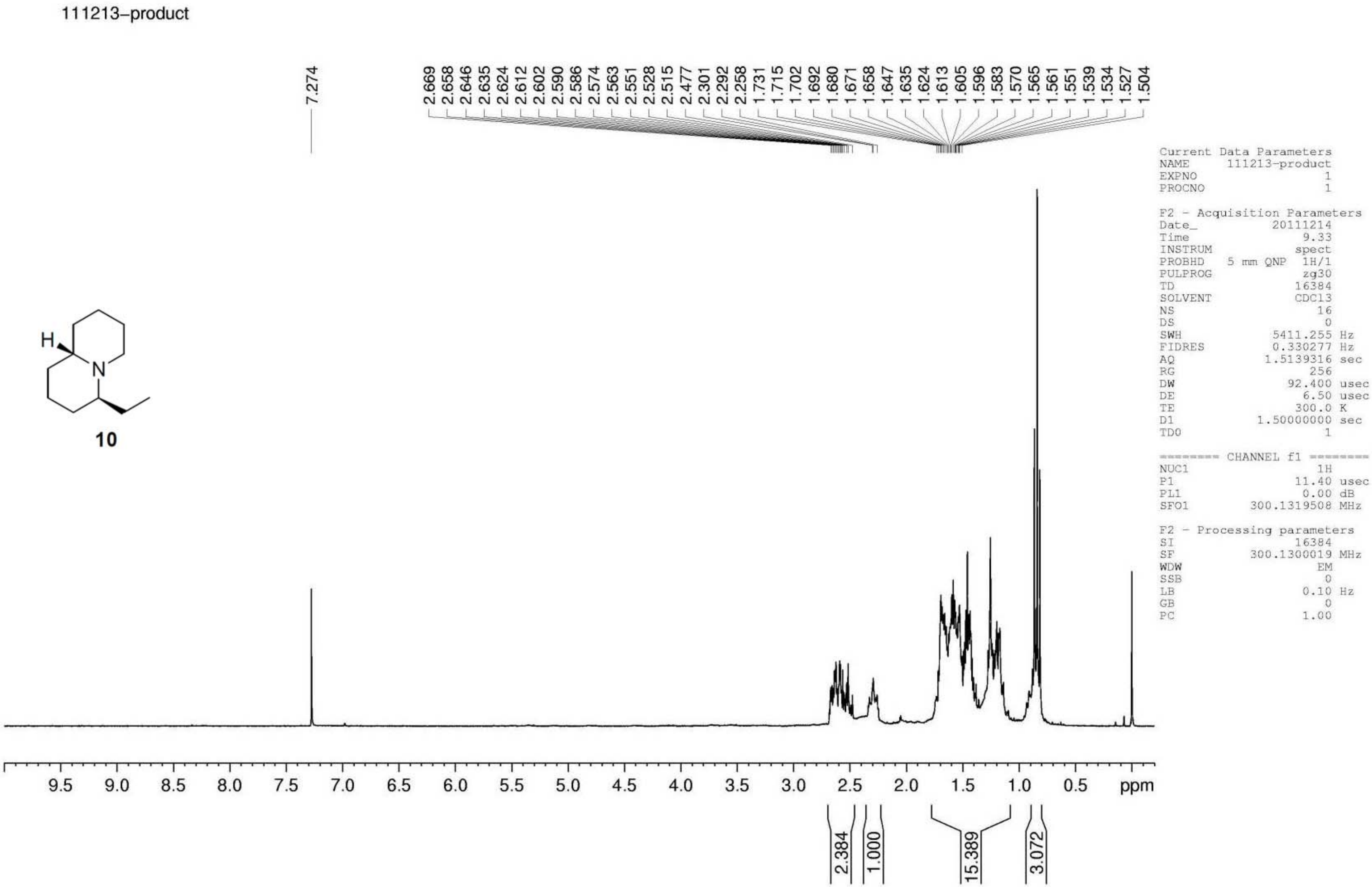
111213–product
7.274
2.669 2.658 2.646 2.635 2.624 2.612 2.602 2.590 2.586 2.574 2.563 2.551 2.528 2.515 2.477 2.301 2.292 2.258 1.731 1.715 1.702 1.692 1.680 1.671 1.658 1.647 1.635 1.624 1.613 1.605 1.596 1.583 1.570 1.565 1.561 1.551 1.539 1.534 1.527 1.504
H
N
10
Current Data Parameters
NAME 111213-product
EXPNO 1
PROCNO 1
F2 - Acquisition Parameters
Date_ 20111214
Time 9.33
INSTRUM spect
PROBHD 5 mm QNP 1H/1
PULPROG zg30
TD 16384
SOLVENT CDCl3
NS 16
DS 0
SWH 5411.255 Hz
FIDRES 0.330277 Hz
AQ 1.5139316 sec
RG 256
DW 92.400 usec
DE 6.50 usec
TE 300.0 K
D1 1.50000000 sec
TD0 1
======== CHANNEL f1 ========
NUC1 1H
P1 11.40 usec
PL1 0.00 dB
SFO1 300.1319508 MHz
F2 - Processing parameters
SI 16384
SF 300.1300019 MHz
WDW EM
SSB 0
LB 0.10 Hz
GB 0
PC 1.00
9.5 9.0 8.5 8.0 7.5 7.0 6.5 6.0 5.5 5.0 4.5 4.0 3.5 3.0 2.5 2.0 1.5 1.0 0.5 ppm
2.384
1.000
15.389
3.072

111213−product

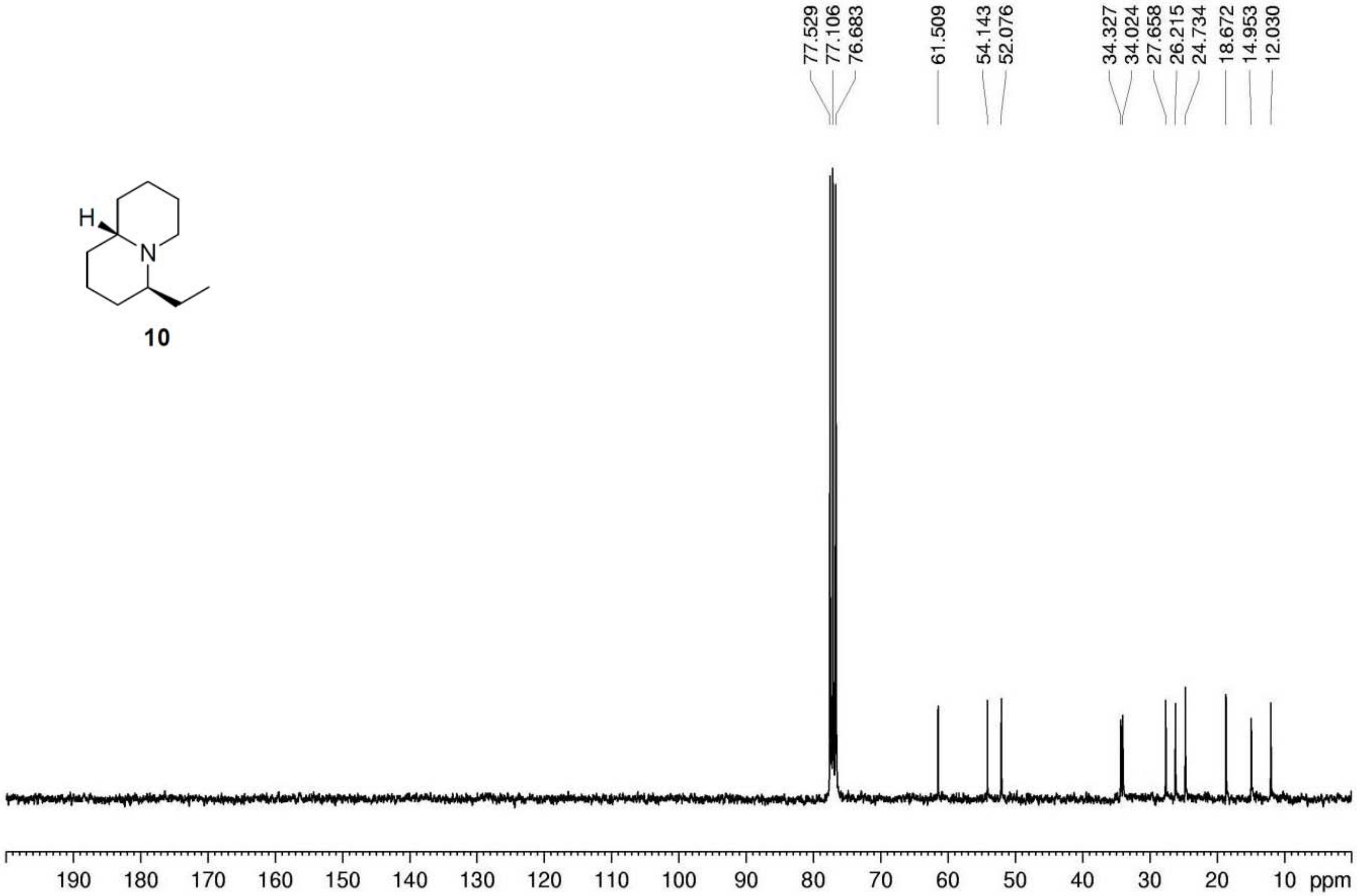


```
Current Data Parameters
NAME       111213-product
EXPNO                   2
PROCNO                  1

F2 - Acquisition Parameters
Date_            20111214
Time                10.06
INSTRUM             spect
PROBHD    5 mm QNP  1H/1
PULPROG           zgpg30
TD                 65536
SOLVENT            CDCl3
NS                   512
DS                     0
SWH            19267.822 Hz
FIDRES          0.294004 Hz
AQ             1.7007092 sec
RG                  8192
DW                25.950 usec
DE                  6.50 usec
TE                 300.0 K
D1            2.00000000 sec
D11           0.03000000 sec
TD0                    1

======== CHANNEL f1 ========
NUC1                 13C
P1                  9.50 usec
PL1                -1.50 dB
SFO1          75.4771825 MHz

======== CHANNEL f2 ========
CPDPRG2          waltz16
NUC2                  1H
PCPD2              90.00 usec
PL2                 0.00 dB
PL12               18.00 dB
PL13               21.00 dB
SFO2         300.1313506 MHz

F2 - Processing parameters
SI                 32768
SF            75.4677424 MHz
WDW                   EM
SSB                    0
LB                  3.00 Hz
GB                     0
PC                  1.00
```

111005–product

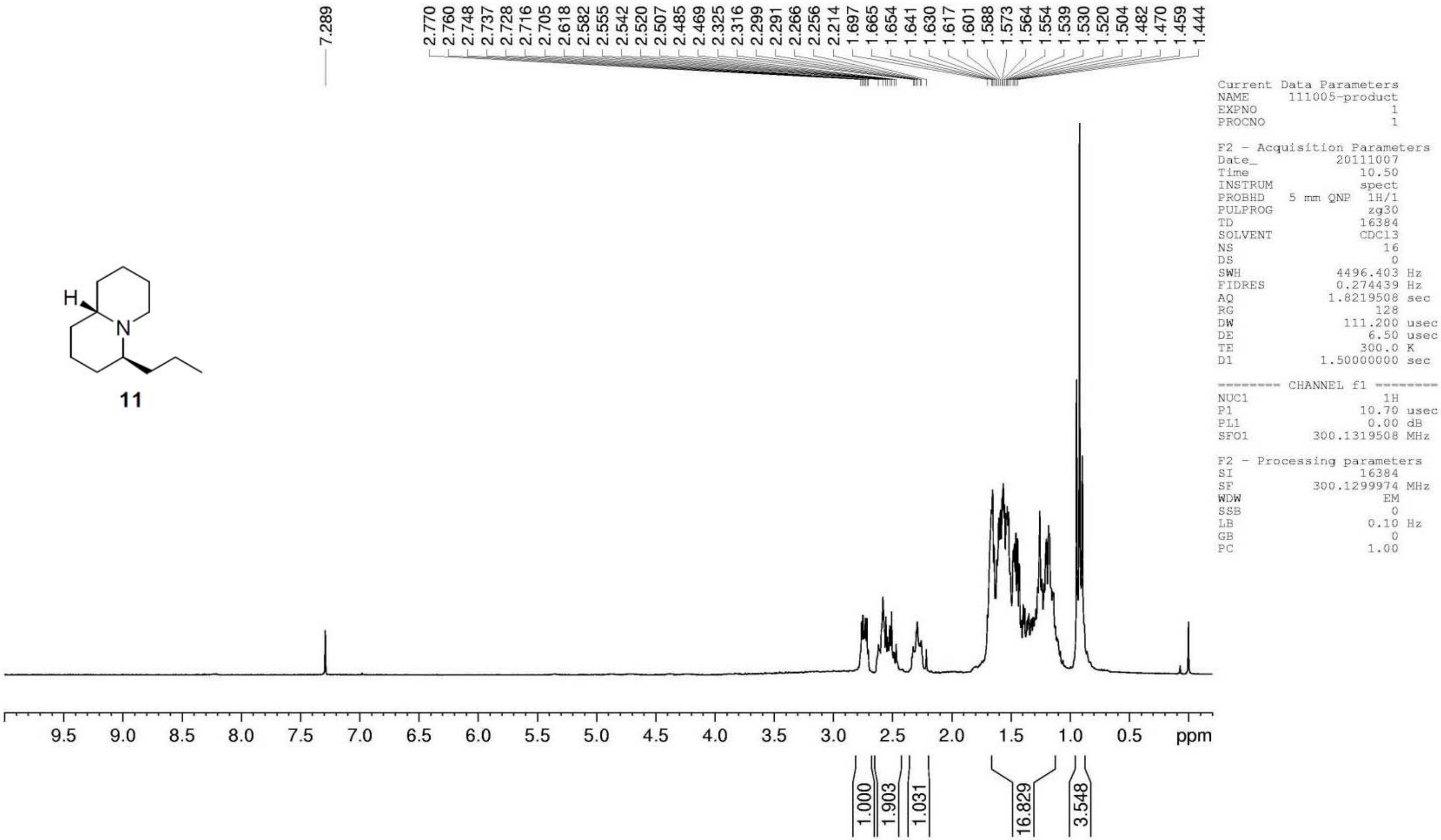

11

111005–product–C13

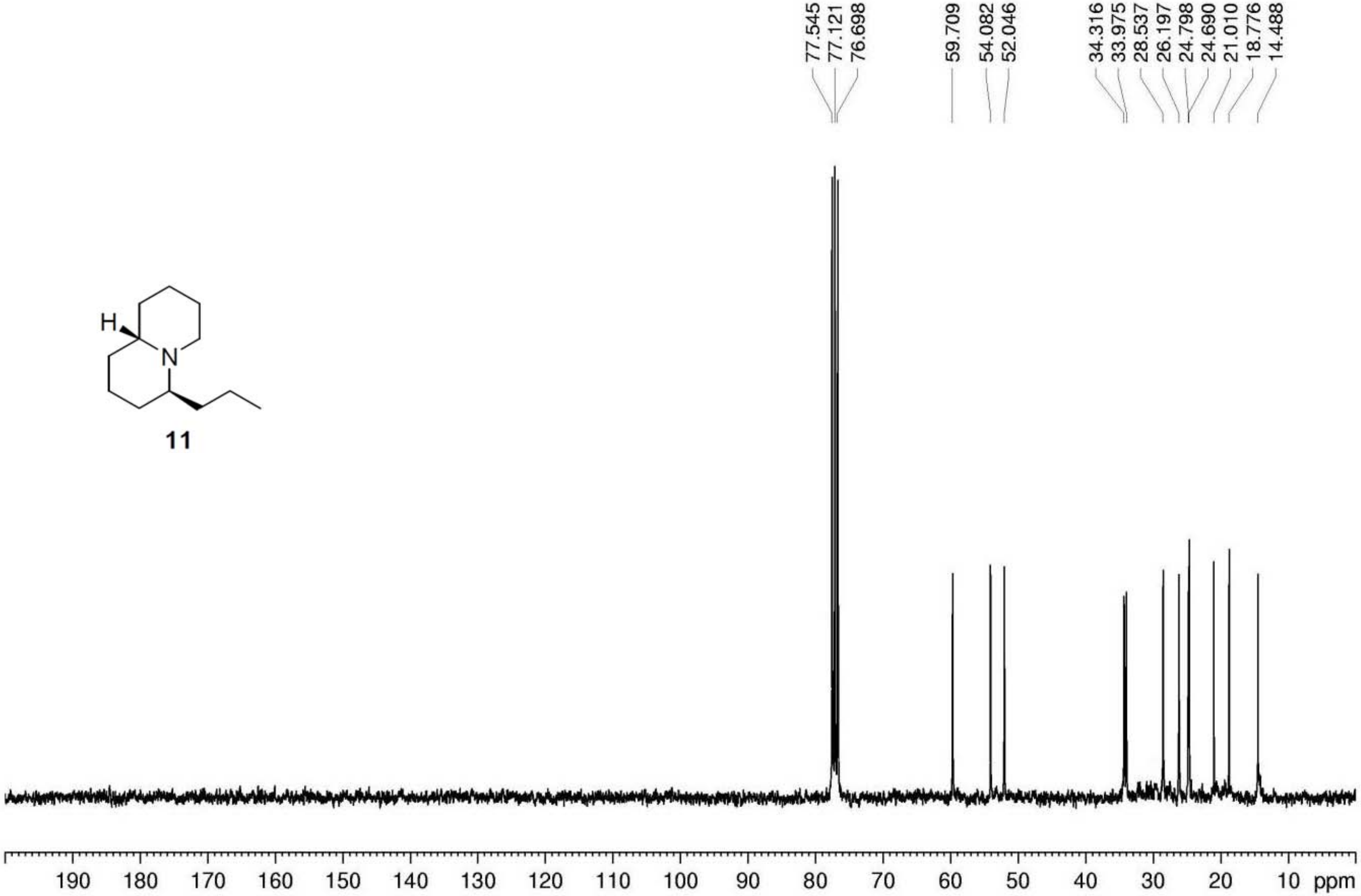


```
Current Data Parameters
NAME       111005-product
EXPNO                   2
PROCNO                  1

F2 - Acquisition Parameters
Date_            20111007
Time                11.17
INSTRUM             spect
PROBHD    5 mm QNP  1H/1
PULPROG           zgpg30
TD                 65536
SOLVENT            CDCl3
NS                   512
DS                     0
SWH            18832.393 Hz
FIDRES          0.287360 Hz
AQ             1.7400308 sec
RG                 13004
DW                26.550 usec
DE                  6.50 usec
TE                 300.0 K
D1            1.20000005 sec
d11           0.03000000 sec
d12           0.00002000 sec

======== CHANNEL f1 ========
NUC1                 13C
P1                 10.10 usec
PL1                 0.00 dB
SFO1          75.4763978 MHz

======== CHANNEL f2 ========
CPDPRG2          waltz16
NUC2                  1H
PCPD2              90.00 usec
PL2                 0.00 dB
PL12               18.10 dB
PL13               21.10 dB
SFO2         300.1313506 MHz

F2 - Processing parameters
SI                 32768
SF            75.4677418 MHz
WDW                   EM
SSB                    0
LB                  3.00 Hz
GB                     0
PC                  1.00
```

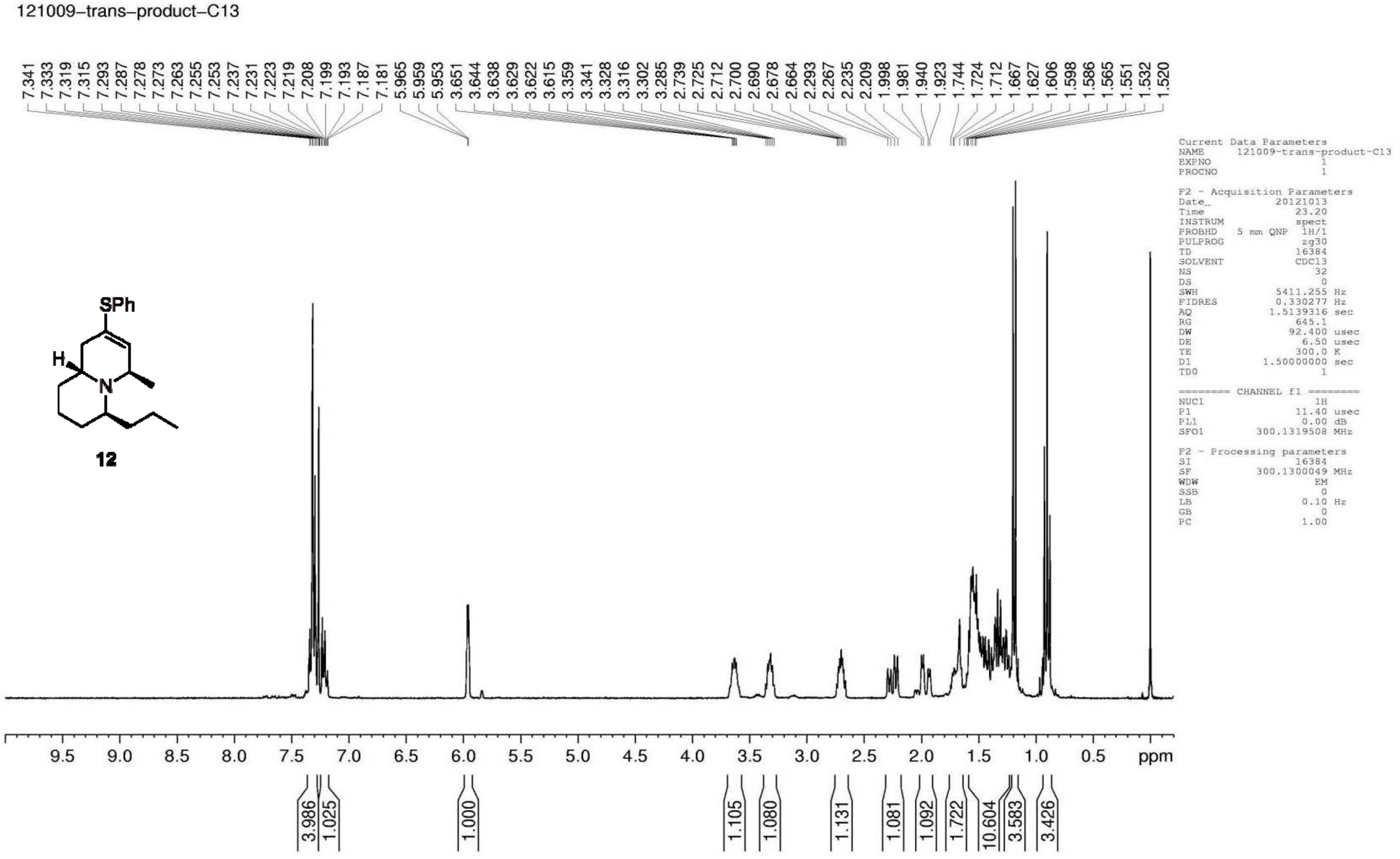
121009-trans-product-C13
7.341 7.333 7.319 7.315 7.293 7.287 7.278 7.273 7.263 7.255 7.253 7.237 7.231 7.223 7.219 7.208 7.199 7.193 7.187 7.181 5.965 5.959 5.953 3.651 3.644 3.638 3.629 3.622 3.615 3.359 3.341 3.328 3.316 3.302 3.285 2.739 2.725 2.712 2.700 2.690 2.678 2.664 2.293 2.267 2.235 2.209 1.998 1.981 1.940 1.923 1.744 1.724 1.712 1.667 1.627 1.606 1.598 1.586 1.565 1.551 1.532 1.520
SPh
H
N
12
Current Data Parameters
NAME 121009-trans-product-C13
EXPNO 1
PROCNO 1
F2 - Acquisition Parameters
Date_ 20121013
Time 23.20
INSTRUM spect
PROBHD 5 mm QNP 1H/1
PULPROG zg30
TD 16384
SOLVENT CDCl3
NS 32
DS 0
SWH 5411.255 Hz
FIDRES 0.330277 Hz
AQ 1.5139316 sec
RG 645.1
DW 92.400 usec
DE 6.50 usec
TE 300.0 K
D1 1.50000000 sec
TD0 1
======== CHANNEL f1 ========
NUC1 1H
P1 11.40 usec
PL1 0.00 dB
SFO1 300.1319508 MHz
F2 - Processing parameters
SI 16384
SF 300.1300049 MHz
WDW EM
SSB 0
LB 0.10 Hz
GB 0
PC 1.00
9.5 9.0 8.5 8.0 7.5 7.0 6.5 6.0 5.5 5.0 4.5 4.0 3.5 3.0 2.5 2.0 1.5 1.0 0.5 ppm
3.986 1.025 1.000 1.105 1.080 1.131 1.081 1.092 1.722 10.604 3.583 3.426

121009–trans–product–C13

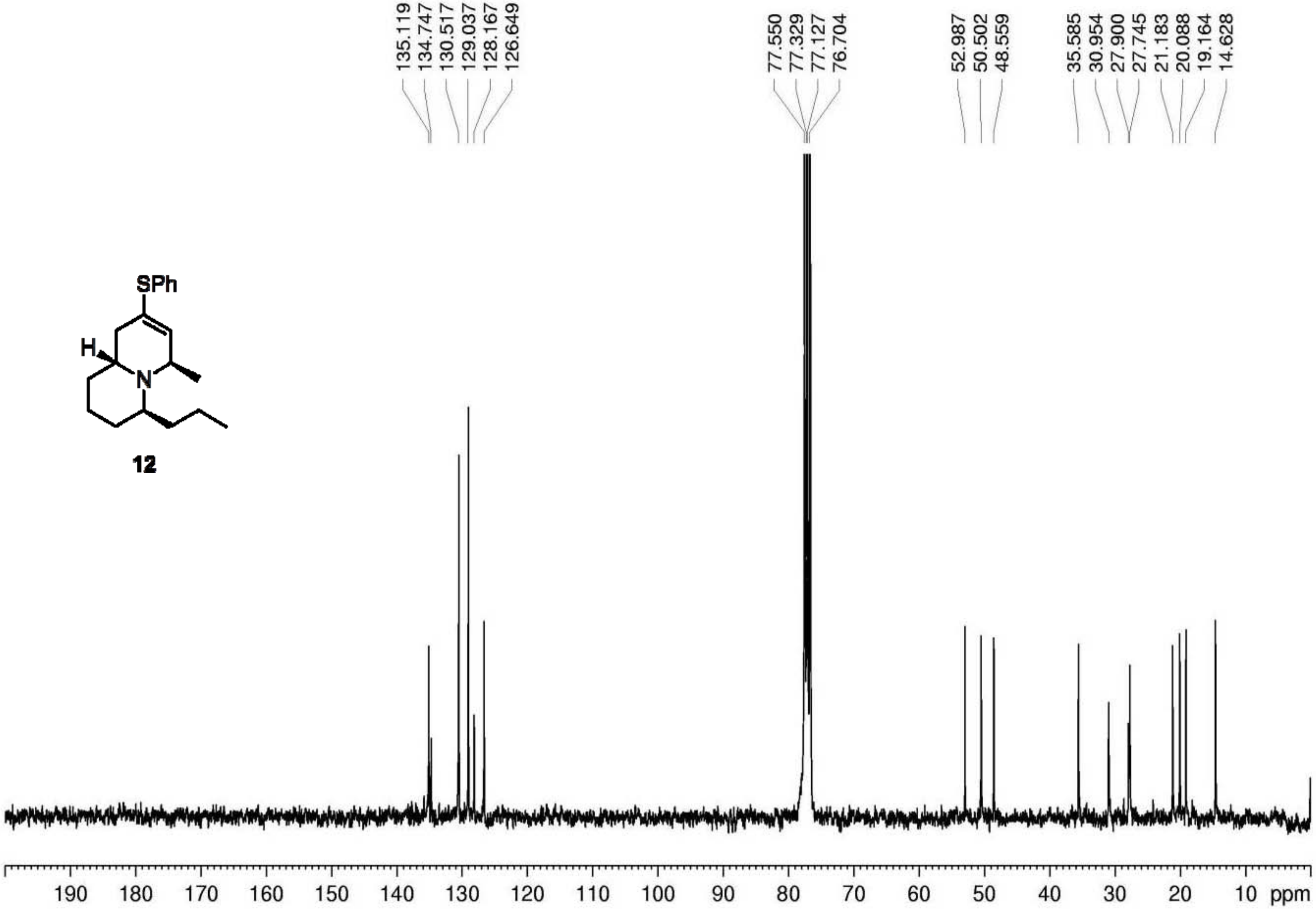

12

```
Current Data Parameters
NAME      121009-trans-product-C13
EXPNO                        2
PROCNO                       1

F2 - Acquisition Parameters
Date_                 20121014
Time                      4.43
INSTRUM                  spect
PROBHD   5 mm QNP  1H/1
PULPROG                zgpg30
TD                       65536
SOLVENT                  CDCl3
NS                        5120
DS                           0
SWH                  19267.822 Hz
FIDRES                0.294004 Hz
AQ                   1.7007092 sec
RG                     14596.5
DW                      25.950 usec
DE                        6.50 usec
TE                       300.0 K
D1                  2.00000000 sec
D11                 0.03000000 sec
TD0                          1

======== CHANNEL f1 ========
NUC1                       13C
P1                        9.50 usec
PL1                      -1.50 dB
SFO1                75.4771825 MHz

======== CHANNEL f2 ========
CPDPRG2                waltz16
NUC2                        1H
PCPD2                    90.00 usec
PL2                       0.00 dB
PL12                     18.00 dB
PL13                     21.00 dB
SFO2               300.1313506 MHz

F2 - Processing parameters
SI                       32768
SF                  75.4677400 MHz
WDW                         EM
SSB                          0
LB                        3.00 Hz
GB                           0
PC                        1.00
```

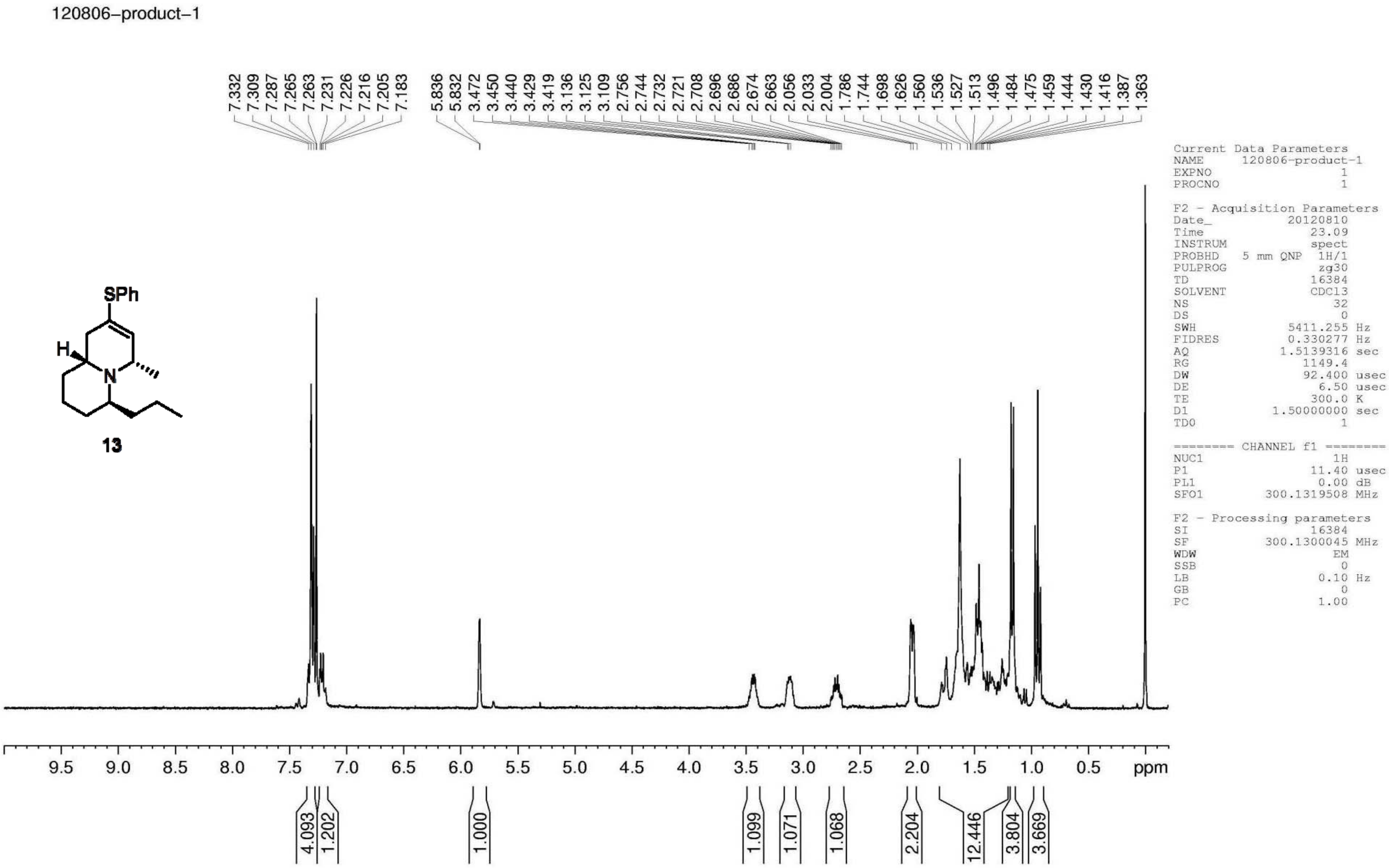
120806−product−1
7.332
7.309
7.287
7.265
7.263
7.231
7.226
7.216
7.205
7.183
5.836
5.832
3.472
3.450
3.440
3.429
3.419
3.136
3.125
3.109
2.756
2.744
2.732
2.721
2.708
2.696
2.686
2.674
2.663
2.056
2.033
2.004
1.786
1.744
1.698
1.626
1.560
1.536
1.527
1.513
1.496
1.484
1.475
1.459
1.444
1.430
1.416
1.387
1.363
SPh
H
N
13
Current Data Parameters
NAME 120806-product-1
EXPNO 1
PROCNO 1
F2 - Acquisition Parameters
Date_ 20120810
Time 23.09
INSTRUM spect
PROBHD 5 mm QNP 1H/1
PULPROG zg30
TD 16384
SOLVENT CDCl3
NS 32
DS 0
SWH 5411.255 Hz
FIDRES 0.330277 Hz
AQ 1.5139316 sec
RG 1149.4
DW 92.400 usec
DE 6.50 usec
TE 300.0 K
D1 1.50000000 sec
TD0 1
======== CHANNEL f1 ========
NUC1 1H
P1 11.40 usec
PL1 0.00 dB
SFO1 300.1319508 MHz
F2 - Processing parameters
SI 16384
SF 300.1300045 MHz
WDW EM
SSB 0
LB 0.10 Hz
GB 0
PC 1.00
9.5 9.0 8.5 8.0 7.5 7.0 6.5 6.0 5.5 5.0 4.5 4.0 3.5 3.0 2.5 2.0 1.5 1.0 0.5 ppm
4.093
1.202
1.000
1.099
1.071
1.068
2.204
12.446
3.804
3.669

120806-product-1

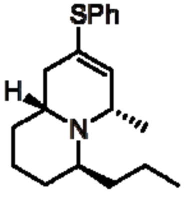

13

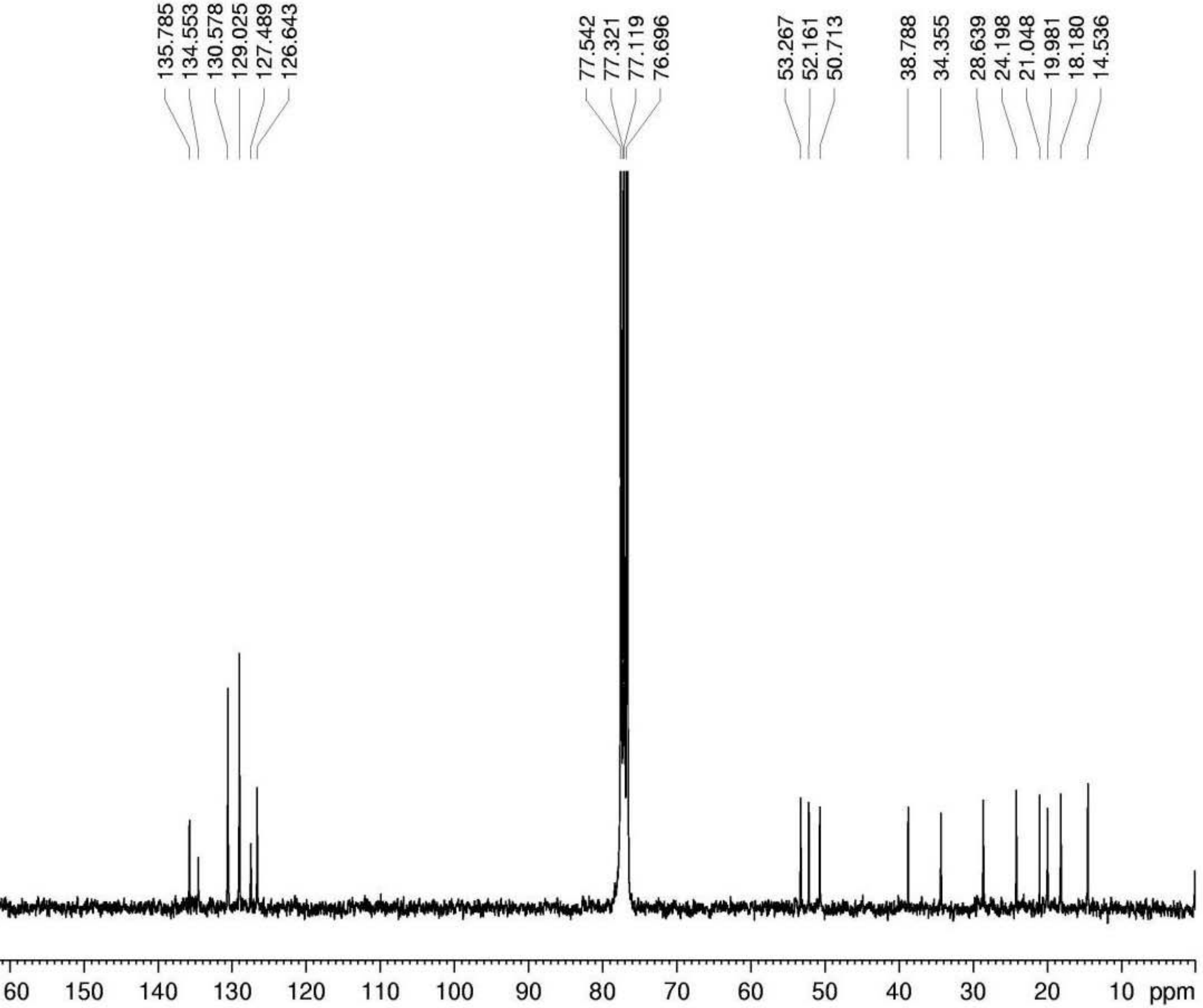


```
Current Data Parameters
NAME      120806-product-1
EXPNO                    2
PROCNO                   1

F2 - Acquisition Parameters
Date_             20120811
Time                  4.32
INSTRUM              spect
PROBHD   5 mm QNP  1H/1
PULPROG            zgpg30
TD                  65536
SOLVENT             CDCl3
NS                   5120
DS                      0
SWH            19267.822 Hz
FIDRES          0.294004 Hz
AQ             1.7007092 sec
RG                11585.2
DW                 25.950 usec
DE                   6.50 usec
TE                  300.0 K
D1          2.00000000 sec
D11         0.03000000 sec
TD0                     1

======== CHANNEL f1 ========
NUC1                  13C
P1                   9.50 usec
PL1                 -1.50 dB
SFO1           75.4771825 MHz

======== CHANNEL f2 ========
CPDPRG2           waltz16
NUC2                   1H
PCPD2               90.00 usec
PL2                  0.00 dB
PL12                18.00 dB
PL13                21.00 dB
SFO2          300.1313506 MHz

F2 - Processing parameters
SI                  32768
SF             75.4677406 MHz
WDW                    EM
SSB                     0
LB                   3.00 Hz
GB                      0
PC                   1.00
```

110824–test–byproduct–CH3

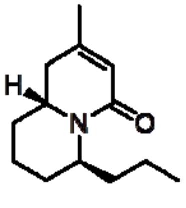

14

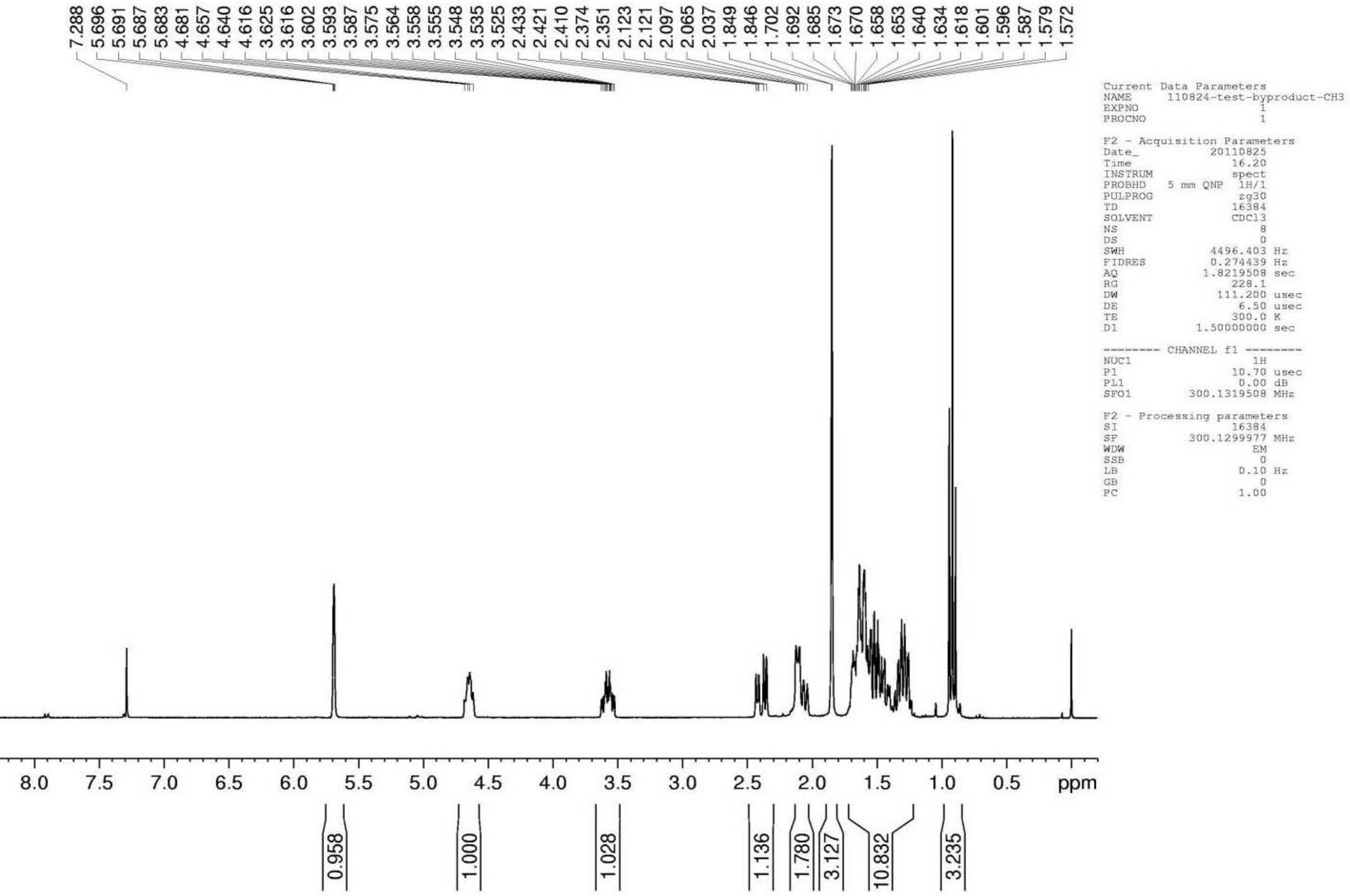

101116-1-C13

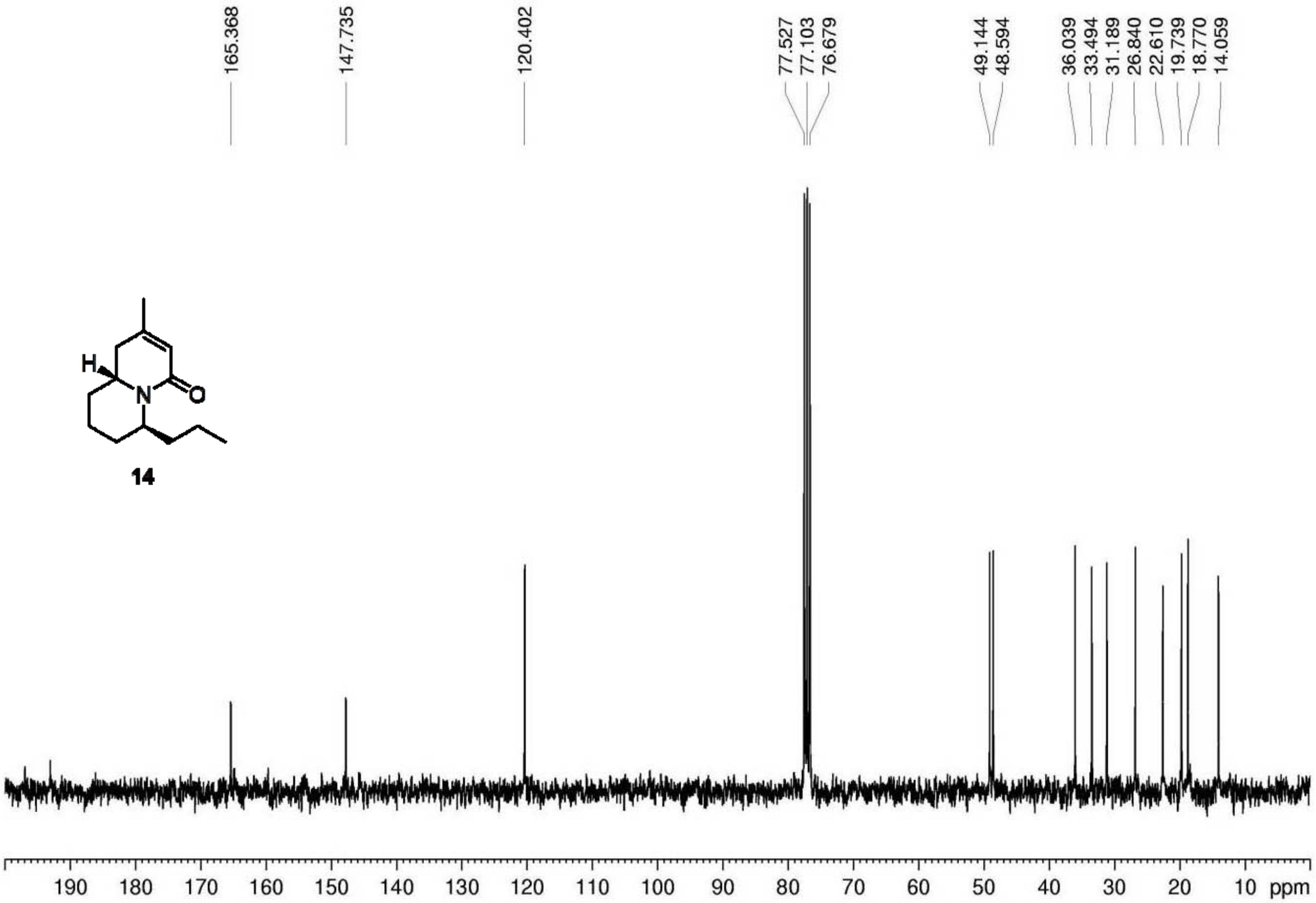

14

```
Current Data Parameters
NAME            101116-1
EXPNO                  2
PROCNO                 1

F2 - Acquisition Parameters
Date_           20101119
Time                0.58
INSTRUM            spect
PROBHD   5 mm QNP  1H/1
PULPROG          zgpg30
TD                65536
SOLVENT           CDCl3
NS                   64
DS                    0
SWH           18832.393 Hz
FIDRES         0.287360 Hz
AQ            1.7400308 sec
RG                13004
DW               26.550 usec
DE                 6.50 usec
TE                300.0 K
D1           1.20000005 sec
d11          0.03000000 sec
d12          0.00002000 sec

======== CHANNEL f1 ========
NUC1                13C
P1                10.10 usec
PL1                0.00 dB
SFO1         75.4763978 MHz

======== CHANNEL f2 ========
CPDPRG2         waltz16
NUC2                 1H
PCPD2             90.00 usec
PL2                0.00 dB
PL12              18.10 dB
PL13              21.10 dB
SFO2        300.1313506 MHz

F2 - Processing parameters
SI                32768
SF           75.4677470 MHz
WDW                  EM
SSB                   0
LB                 3.00 Hz
GB                    0
PC                 1.00
```

121122-product1

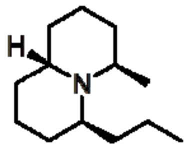

15

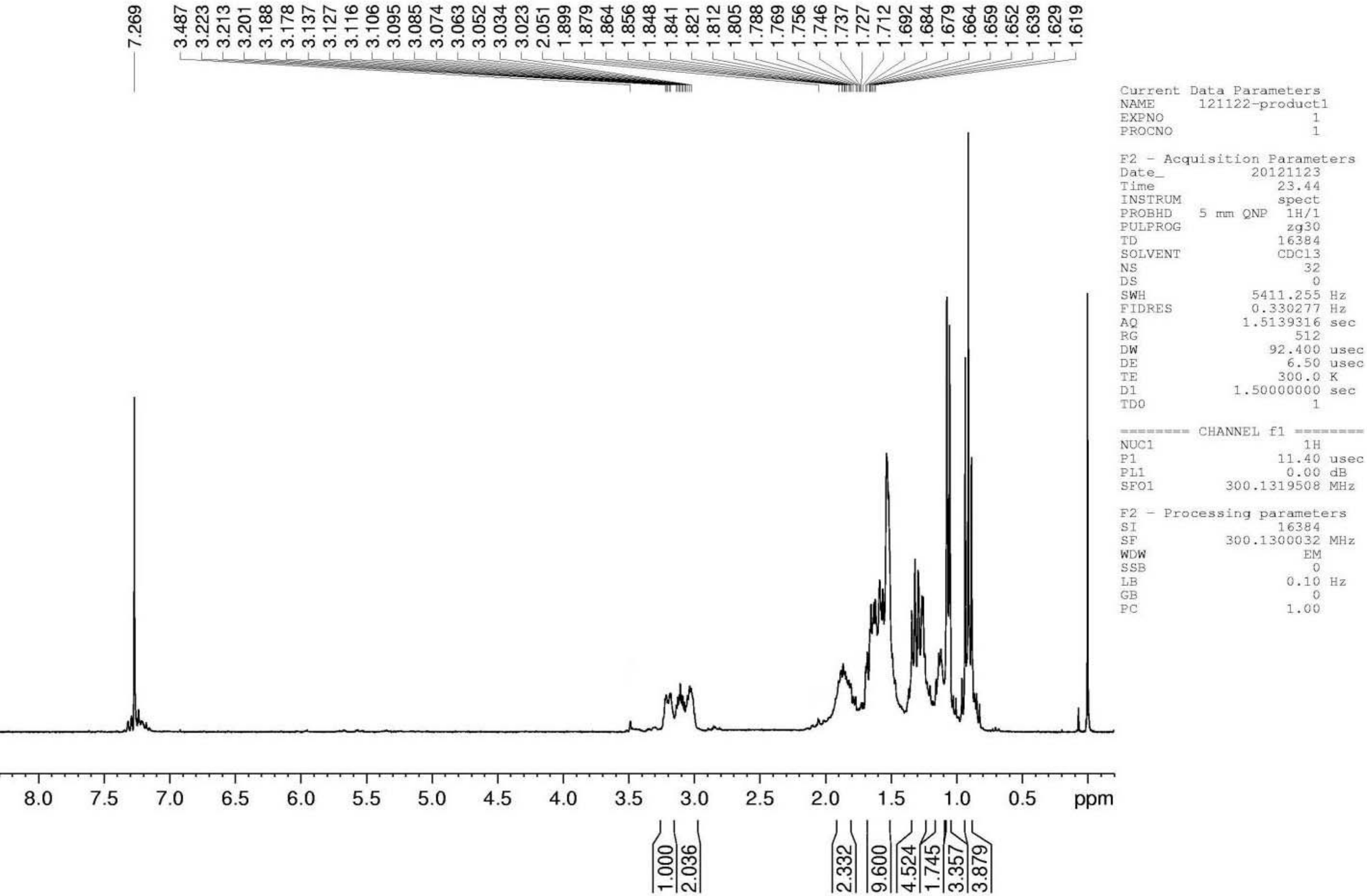

121122-2

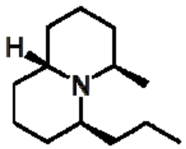

15

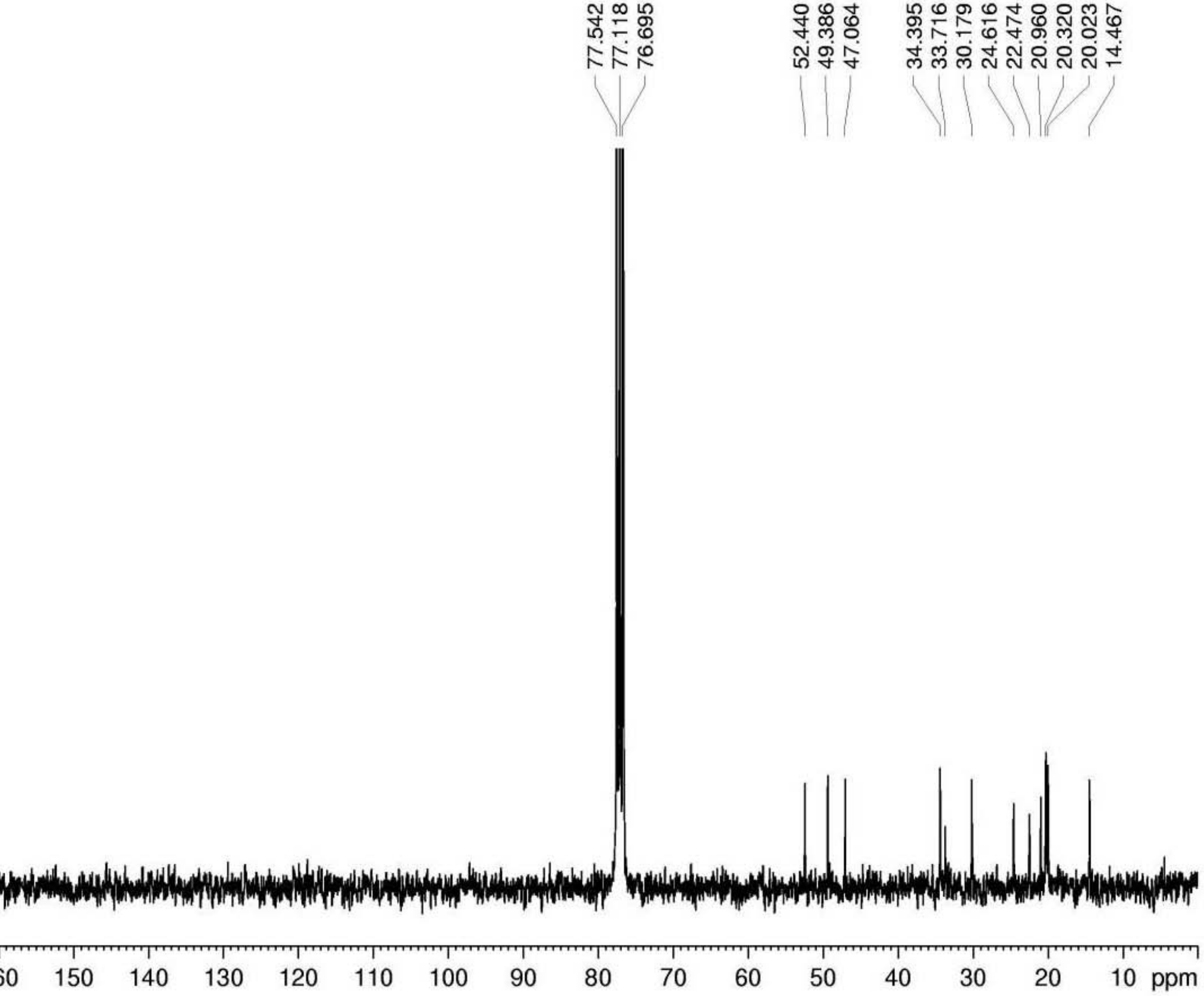


```
Current Data Parameters
NAME              121122-2
EXPNO                    2
PROCNO                   1

F2 - Acquisition Parameters
Date_             20121123
Time                 14.04
INSTRUM              spect
PROBHD   5 mm QNP  1H/1
PULPROG            zgpg30
TD                  65536
SOLVENT             CDCl3
NS                   1024
DS                      0
SWH             19267.822 Hz
FIDRES           0.294004 Hz
AQ              1.7007092 sec
RG                  16384
DW                 25.950 usec
DE                   6.50 usec
TE                  300.0 K
D1             2.00000000 sec
D11            0.03000000 sec
TD0                     1

======== CHANNEL f1 ========
NUC1                  13C
P1                   9.50 usec
PL1                 -1.50 dB
SFO1          75.4771825 MHz

======== CHANNEL f2 ========
CPDPRG2           waltz16
NUC2                   1H
PCPD2               90.00 usec
PL2                  0.00 dB
PL12                18.00 dB
PL13                21.00 dB
SFO2         300.1313506 MHz

F2 - Processing parameters
SI                  32768
SF             75.4677412 MHz
WDW                    EM
SSB                     0
LB                   3.00 Hz
GB                      0
PC                   1.00
```

130416–66–CO2Me–mCPBA

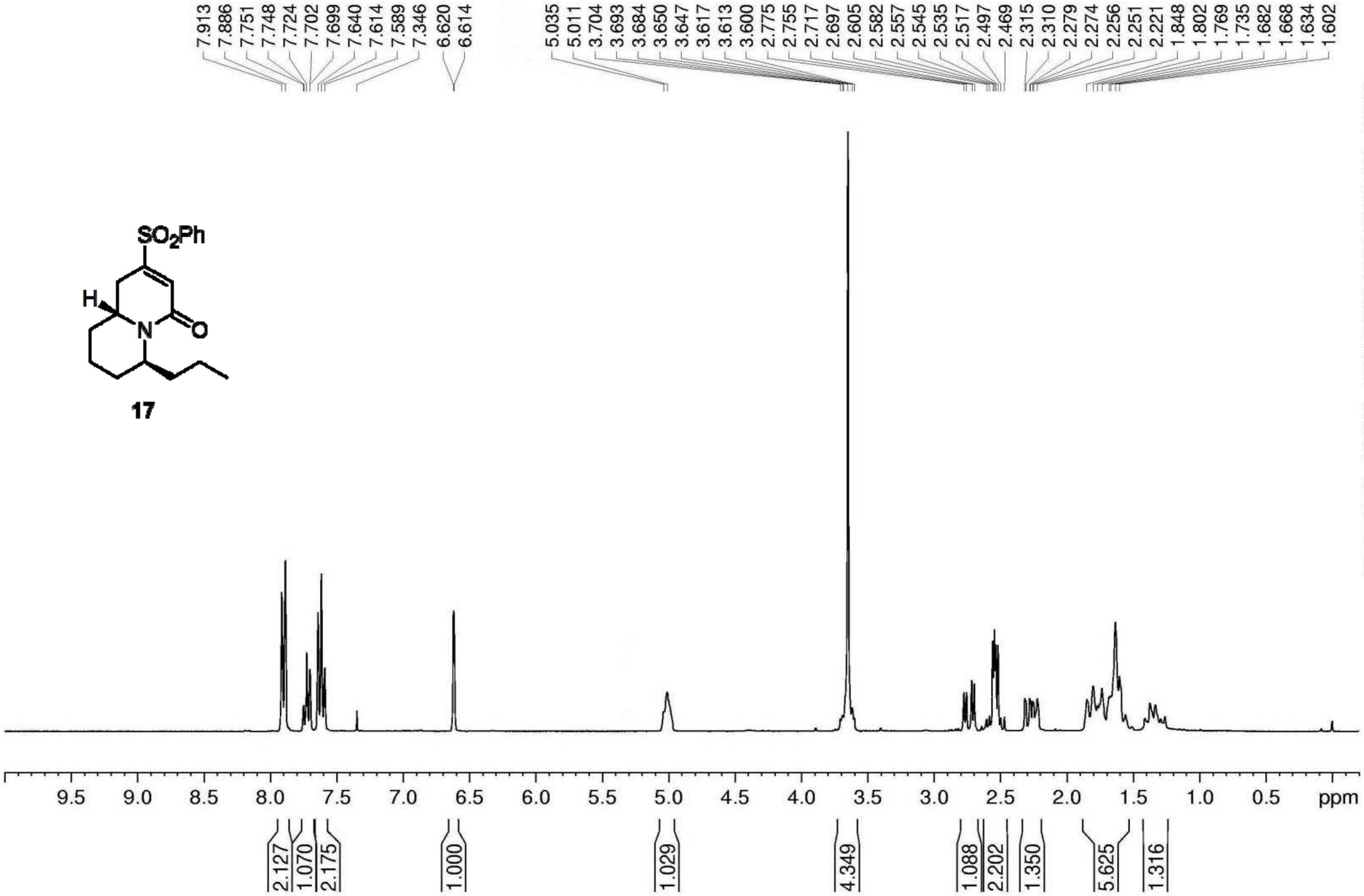


```
Current Data Parameters
NAME      130416-66-CO2Me-mCPBA
EXPNO                         1
PROCNO                        1

F2 - Acquisition Parameters
Date_                  20130416
Time                      14.57
INSTRUM                   spect
PROBHD    5 mm QNP  1H/1
PULPROG                    zg30
TD                        16384
SOLVENT                   CDCl3
NS                           16
DS                            0
SWH                    5411.255 Hz
FIDRES                 0.330277 Hz
AQ                    1.5139316 sec
RG                          128
DW                       92.400 usec
DE                         6.50 usec
TE                        300.0 K
D1                   1.50000000 sec
TD0                           1

======== CHANNEL f1 ========
NUC1                         1H
P1                        11.40 usec
PL1                        0.00 dB
SFO1                300.1319508 MHz

F2 - Processing parameters
SI                        16384
SF                  300.1299794 MHz
WDW                          EM
SSB                           0
LB                         0.10 Hz
GB                            0
PC                         1.00
```

130416-66-CO2Me-mCPBA

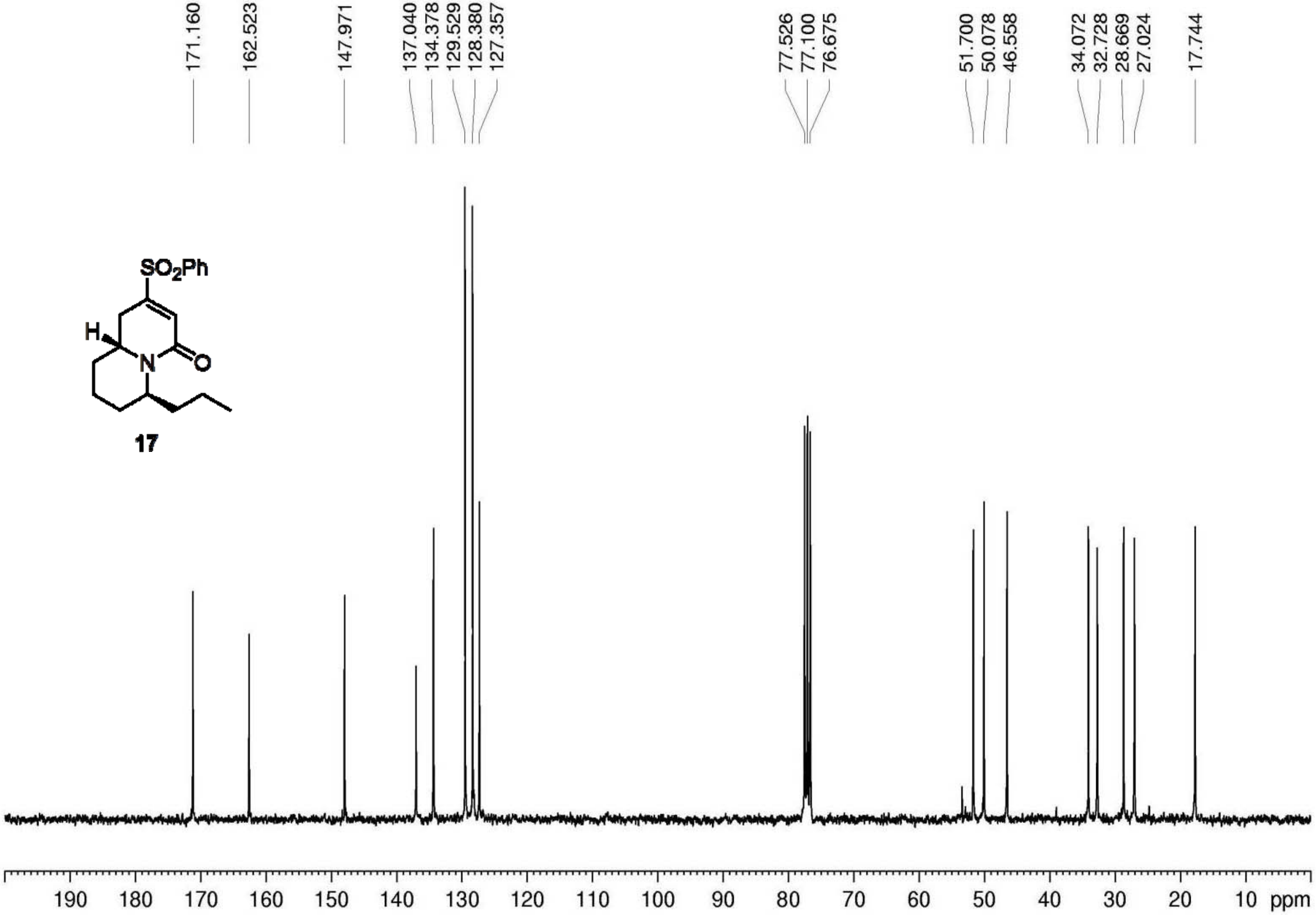


```
Current Data Parameters
NAME     130416-66-CO2Me-mCPBA
EXPNO                     2
PROCNO                    1

F2 - Acquisition Parameters
Date_              20130416
Time                  15.36
INSTRUM               spect
PROBHD     5 mm QNP  1H/1
PULPROG             zgpg30
TD                   65536
SOLVENT              CDCl3
NS                     600
DS                       0
SWH            19267.822 Hz
FIDRES          0.294004 Hz
AQ             1.7007092 sec
RG               14596.5
DW                25.950 usec
DE                  6.50 usec
TE                 300.0 K
D1            2.00000000 sec
D11           0.03000000 sec
TD0                      1

======== CHANNEL f1 ========
NUC1                   13C
P1                    9.50 usec
PL1                  -1.50 dB
SFO1           75.4760505 MHz

======== CHANNEL f2 ========
CPDPRG2            waltz16
NUC2                    1H
PCPD2                90.00 usec
PL2                   0.00 dB
PL12                 18.00 dB
PL13                 21.00 dB
SFO2          300.1313506 MHz

F2 - Processing parameters
SI                   32768
SF             75.4677587 MHz
WDW                     EM
SSB                      0
LB                    3.00 Hz
GB                       0
PC                    1.00
```

110530–product

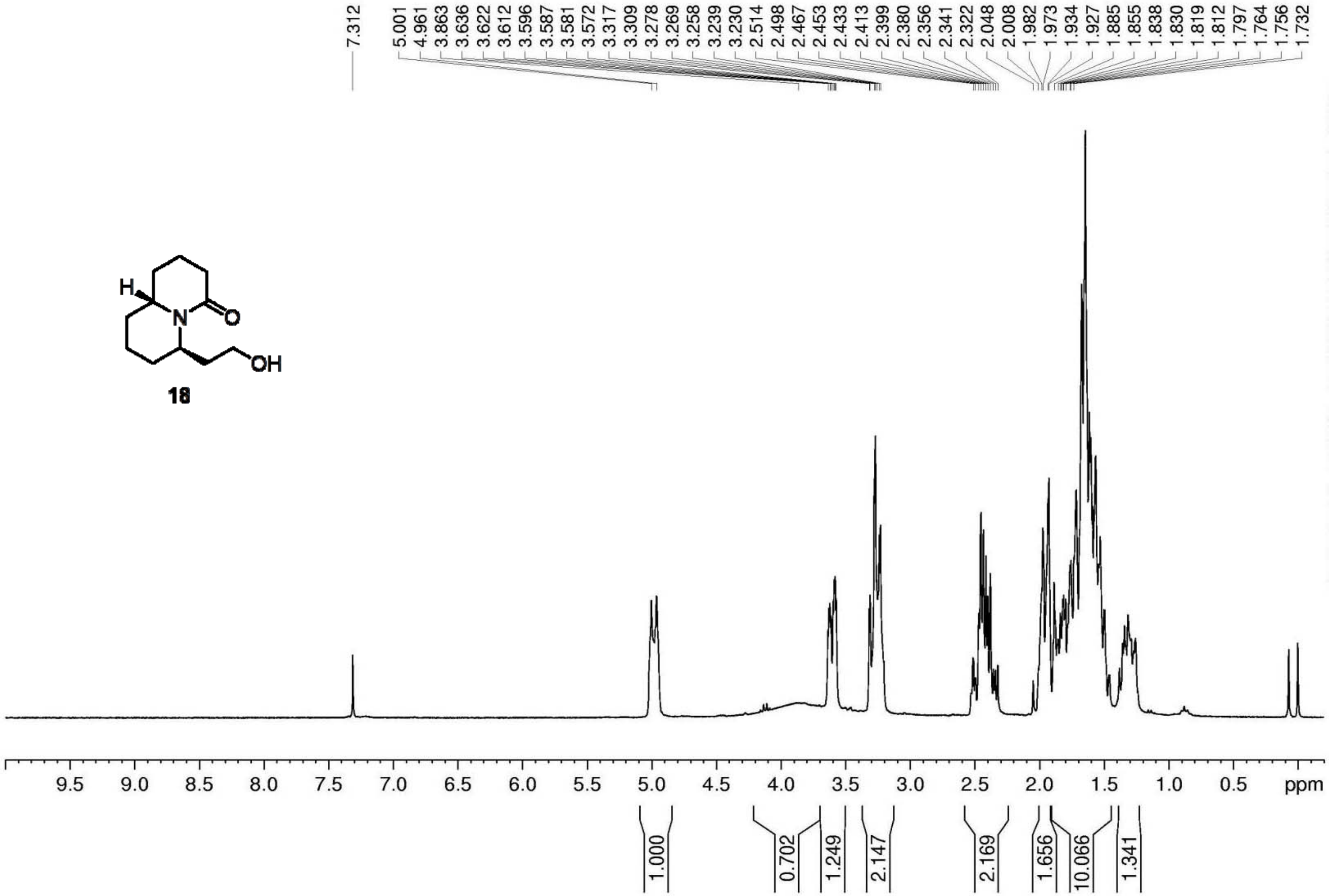


```
Current Data Parameters
NAME      110530-product
EXPNO                  1
PROCNO                 1

F2 - Acquisition Parameters
Date_           20110601
Time                5.17
INSTRUM            spect
PROBHD   5 mm QNP  1H/1
PULPROG             zg30
TD                 16384
SOLVENT            CDCl3
NS                    12
DS                     0
SWH             4496.403 Hz
FIDRES          0.274439 Hz
AQ             1.8219508 sec
RG                   128
DW               111.200 usec
DE                  6.50 usec
TE                 300.0 K
D1            1.50000000 sec

======== CHANNEL f1 ========
NUC1                  1H
P1                 10.70 usec
PL1                 0.00 dB
SFO1         300.1319508 MHz

F2 - Processing parameters
SI                 16384
SF           300.1299903 MHz
WDW                   EM
SSB                    0
LB                  0.10 Hz
GB                     0
PC                  1.00
```

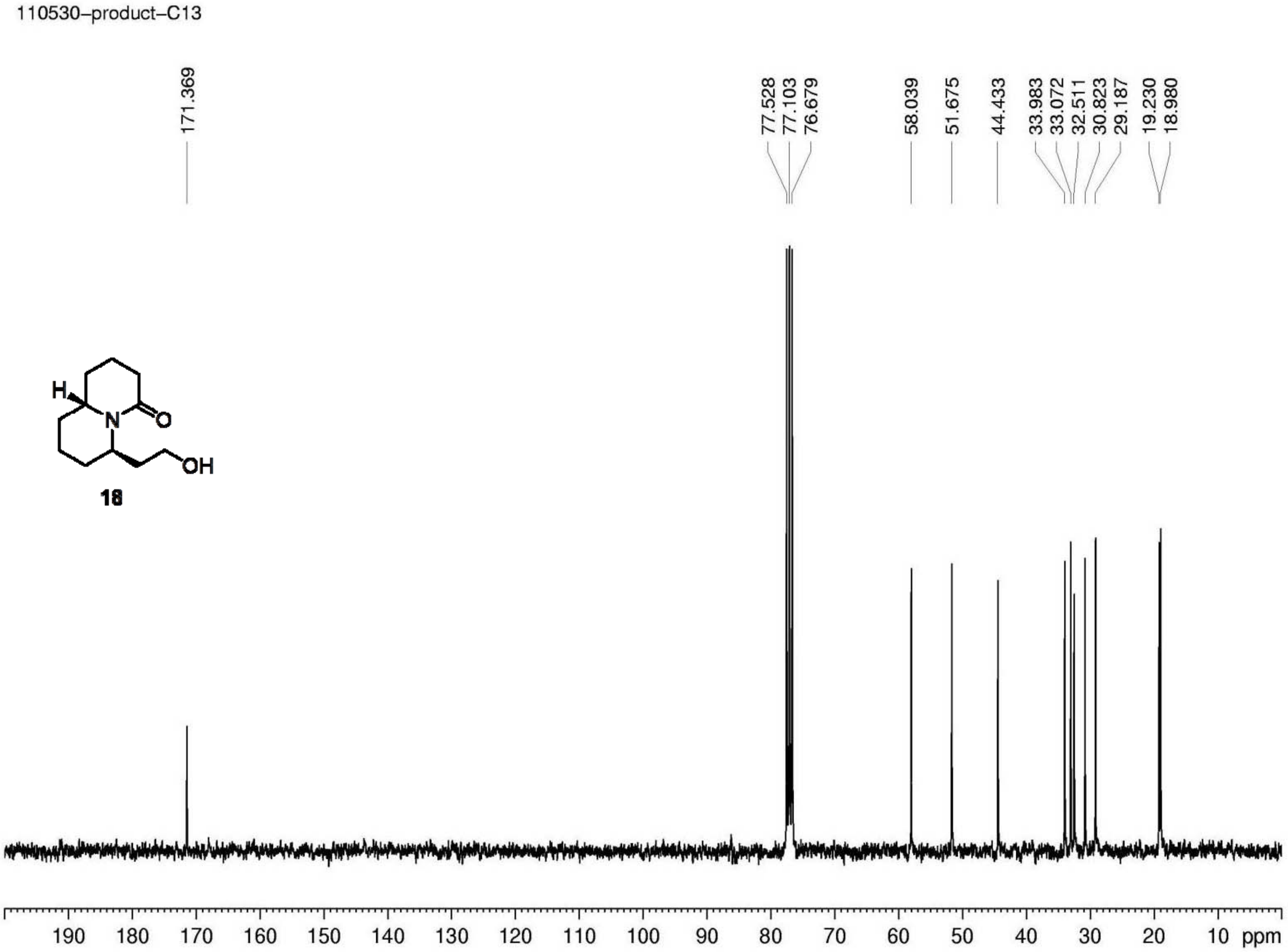

Current Data Parameters
NAME 110530-product
EXPNO 2
PROCNO 1

F2 - Acquisition Parameters
Date_ 20110601
Time 5.32
INSTRUM spect
PROBHD 5 mm QNP 1H/1
PULPROG zgpg30
TD 65536
SOLVENT CDCl3
NS 256
DS 0
SWH 18832.393 Hz
FIDRES 0.287360 Hz
AQ 1.7400308 sec
RG 14596.5
DW 26.550 usec
DE 6.50 usec
TE 300.0 K
D1 1.20000005 sec
d11 0.03000000 sec
d12 0.00002000 sec

======== CHANNEL f1 ========
NUC1 13C
P1 10.10 usec
PL1 0.00 dB
SFO1 75.4763978 MHz

======== CHANNEL f2 ========
CPDPRG2 waltz16
NUC2 1H
PCPD2 90.00 usec
PL2 0.00 dB
PL12 18.10 dB
PL13 21.10 dB
SFO2 300.1313506 MHz

F2 - Processing parameters
SI 32768
SF 75.4677482 MHz
WDW EM
SSB 0
LB 3.00 Hz
GB 0
PC 1.00

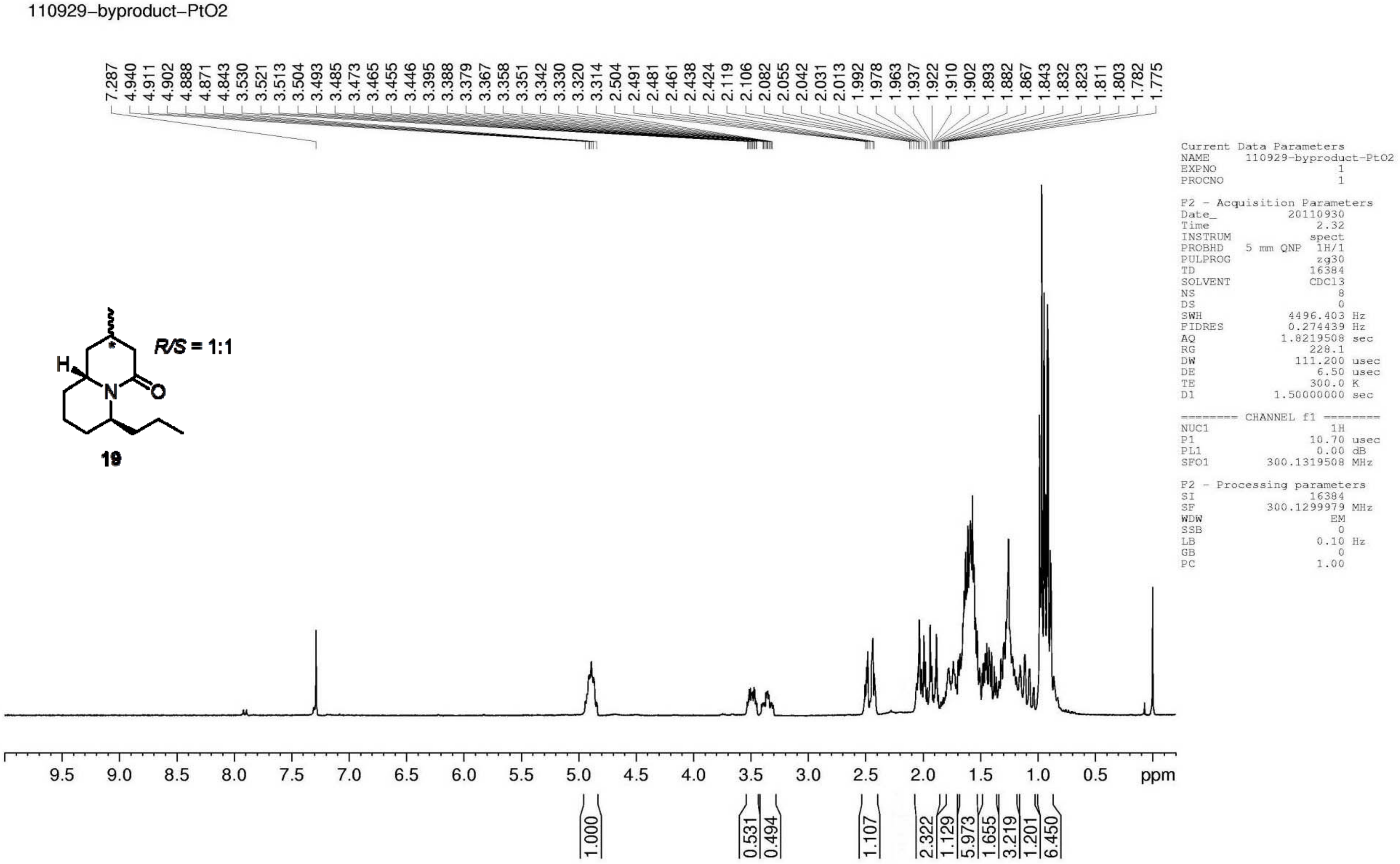
110929-byproduct-PtO2
R/S = 1:1
19
Current Data Parameters
NAME 110929-byproduct-PtO2
EXPNO 1
PROCNO 1
F2 - Acquisition Parameters
Date_ 20110930
Time 2.32
INSTRUM spect
PROBHD 5 mm QNP 1H/1
PULPROG zg30
TD 16384
SOLVENT CDCl3
NS 8
DS 0
SWH 4496.403 Hz
FIDRES 0.274439 Hz
AQ 1.8219508 sec
RG 228.1
DW 111.200 usec
DE 6.50 usec
TE 300.0 K
D1 1.50000000 sec
======== CHANNEL f1 ========
NUC1 1H
P1 10.70 usec
PL1 0.00 dB
SFO1 300.1319508 MHz
F2 - Processing parameters
SI 16384
SF 300.1299979 MHz
WDW EM
SSB 0
LB 0.10 Hz
GB 0
PC 1.00
ppm

110929-byproduct-PtO2-C13

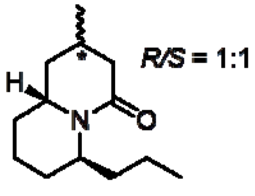

19

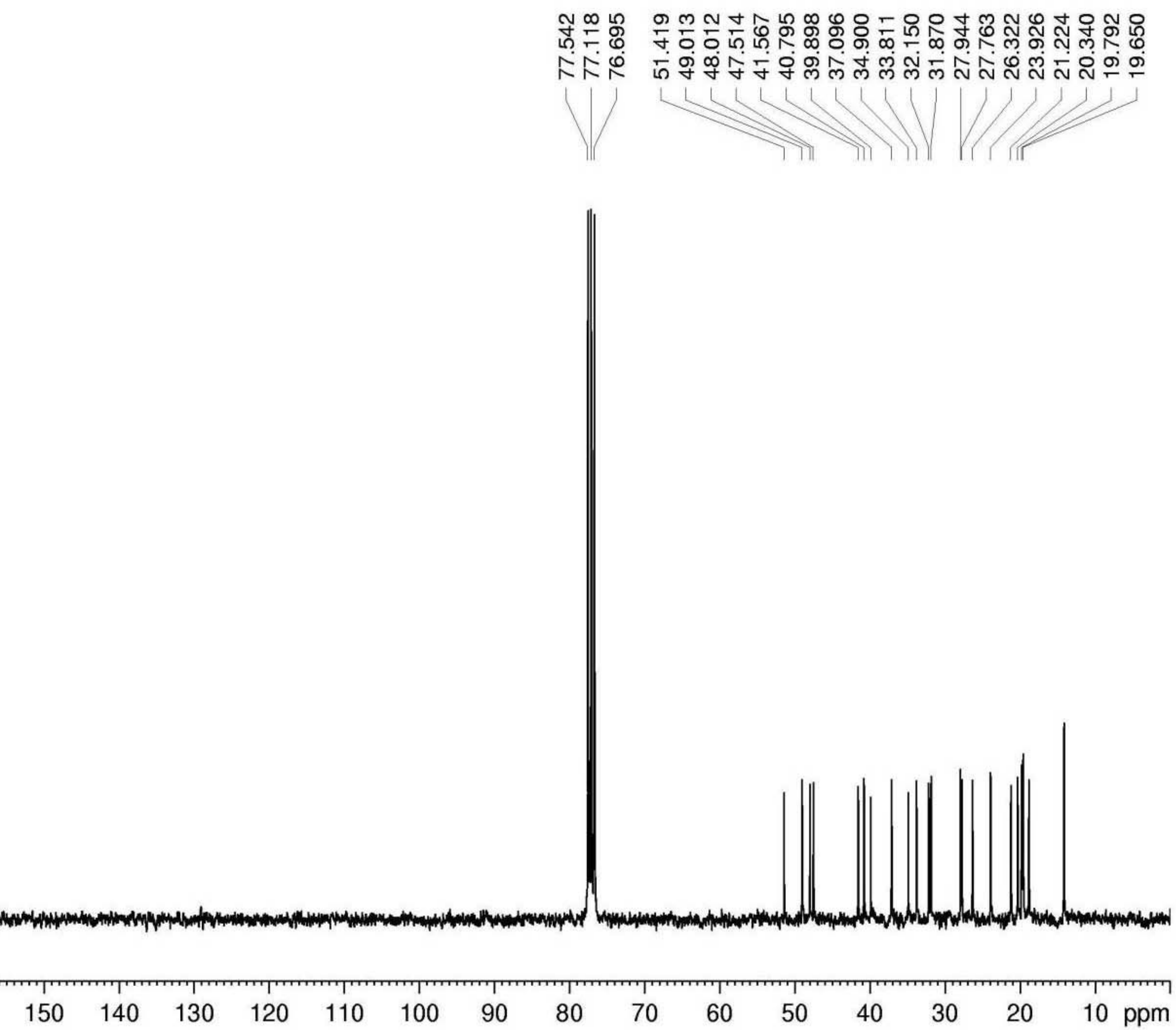


```
Current Data Parameters
NAME      110929-byproduct-PtO2
EXPNO                       2
PROCNO                      1

F2 - Acquisition Parameters
Date_                20110930
Time                     3.24
INSTRUM                 spect
PROBHD    5 mm QNP  1H/1
PULPROG               zgpg30
TD                      65536
SOLVENT                 CDCl3
NS                       1024
DS                          0
SWH                 18832.393 Hz
FIDRES               0.287360 Hz
AQ                  1.7400308 sec
RG                    14596.5
DW                     26.550 usec
DE                       6.50 usec
TE                      300.0 K
D1                 1.20000005 sec
d11                0.03000000 sec
d12                0.00002000 sec

======== CHANNEL f1 ========
NUC1                      13C
P1                      10.10 usec
PL1                      0.00 dB
SFO1               75.4763978 MHz

======== CHANNEL f2 ========
CPDPRG2               waltz16
NUC2                       1H
PCPD2                   90.00 usec
PL2                      0.00 dB
PL12                    18.10 dB
PL13                    21.10 dB
SFO2              300.1313506 MHz

F2 - Processing parameters
SI                      32768
SF                 75.4677430 MHz
WDW                        EM
SSB                         0
LB                       3.00 Hz
GB                          0
PC                       1.00
```

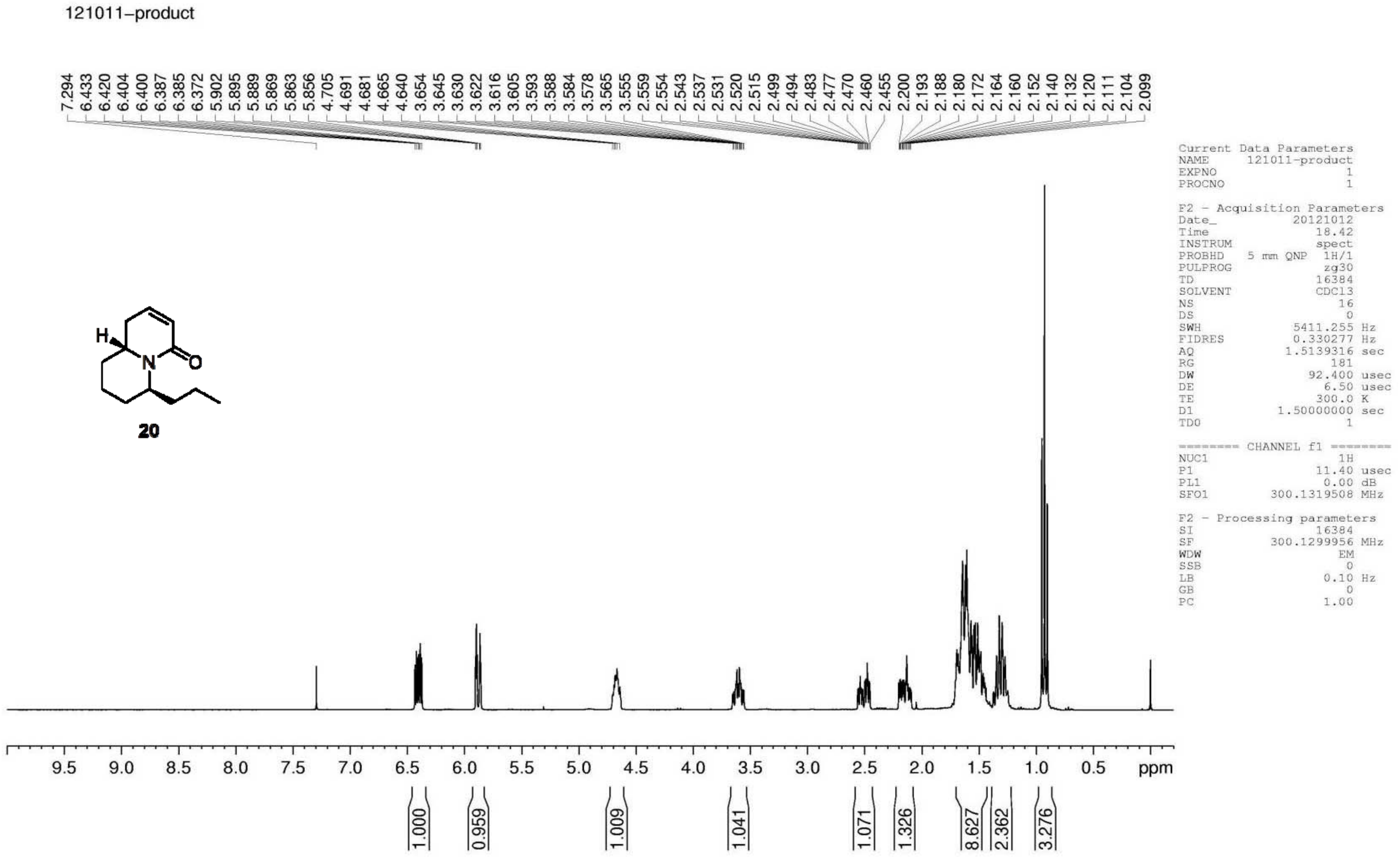
121011−product
7.294
6.433
6.420
6.404
6.400
6.387
6.385
6.372
5.902
5.895
5.889
5.869
5.863
5.856
4.705
4.691
4.681
4.665
4.640
3.654
3.645
3.630
3.622
3.616
3.605
3.593
3.588
3.584
3.578
3.565
3.555
2.559
2.554
2.543
2.537
2.531
2.520
2.515
2.499
2.494
2.483
2.477
2.470
2.460
2.455
2.200
2.193
2.188
2.180
2.172
2.164
2.160
2.152
2.140
2.132
2.120
2.111
2.104
2.099
H
N
O
20
Current Data Parameters
NAME 121011-product
EXPNO 1
PROCNO 1
F2 - Acquisition Parameters
Date_ 20121012
Time 18.42
INSTRUM spect
PROBHD 5 mm QNP 1H/1
PULPROG zg30
TD 16384
SOLVENT CDCl3
NS 16
DS 0
SWH 5411.255 Hz
FIDRES 0.330277 Hz
AQ 1.5139316 sec
RG 181
DW 92.400 usec
DE 6.50 usec
TE 300.0 K
D1 1.50000000 sec
TD0 1
======== CHANNEL f1 ========
NUC1 1H
P1 11.40 usec
PL1 0.00 dB
SFO1 300.1319508 MHz
F2 - Processing parameters
SI 16384
SF 300.1299956 MHz
WDW EM
SSB 0
LB 0.10 Hz
GB 0
PC 1.00
9.5
9.0
8.5
8.0
7.5
7.0
6.5
6.0
5.5
5.0
4.5
4.0
3.5
3.0
2.5
2.0
1.5
1.0
0.5
ppm
1.000
0.959
1.009
1.041
1.071
1.326
8.627
2.362
3.276

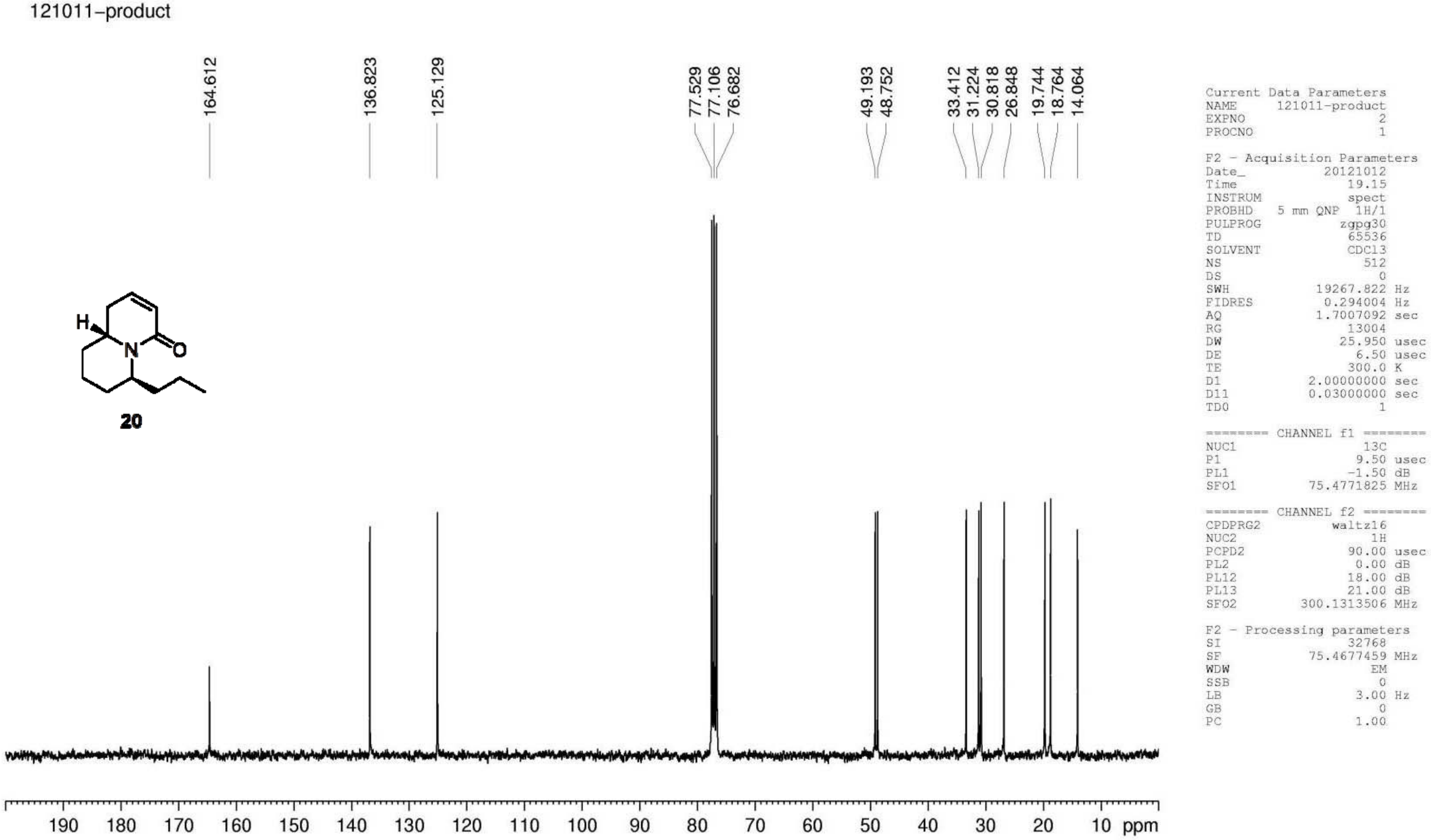
121011-product
164.612
136.823
125.129
77.529
77.106
76.682
49.193
48.752
33.412
31.224
30.818
26.848
19.744
18.764
14.064
20
190 180 170 160 150 140 130 120 110 100 90 80 70 60 50 40 30 20 10 ppm
Current Data Parameters
NAME 121011-product
EXPNO 2
PROCNO 1
F2 - Acquisition Parameters
Date_ 20121012
Time 19.15
INSTRUM spect
PROBHD 5 mm QNP 1H/1
PULPROG zgpg30
TD 65536
SOLVENT CDCl3
NS 512
DS 0
SWH 19267.822 Hz
FIDRES 0.294004 Hz
AQ 1.7007092 sec
RG 13004
DW 25.950 usec
DE 6.50 usec
TE 300.0 K
D1 2.00000000 sec
D11 0.03000000 sec
TD0 1
======== CHANNEL f1 ========
NUC1 13C
P1 9.50 usec
PL1 -1.50 dB
SFO1 75.4771825 MHz
======== CHANNEL f2 ========
CPDPRG2 waltz16
NUC2 1H
PCPD2 90.00 usec
PL2 0.00 dB
PL12 18.00 dB
PL13 21.00 dB
SFO2 300.1313506 MHz
F2 - Processing parameters
SI 32768
SF 75.4677459 MHz
WDW EM
SSB 0
LB 3.00 Hz
GB 0
PC 1.00

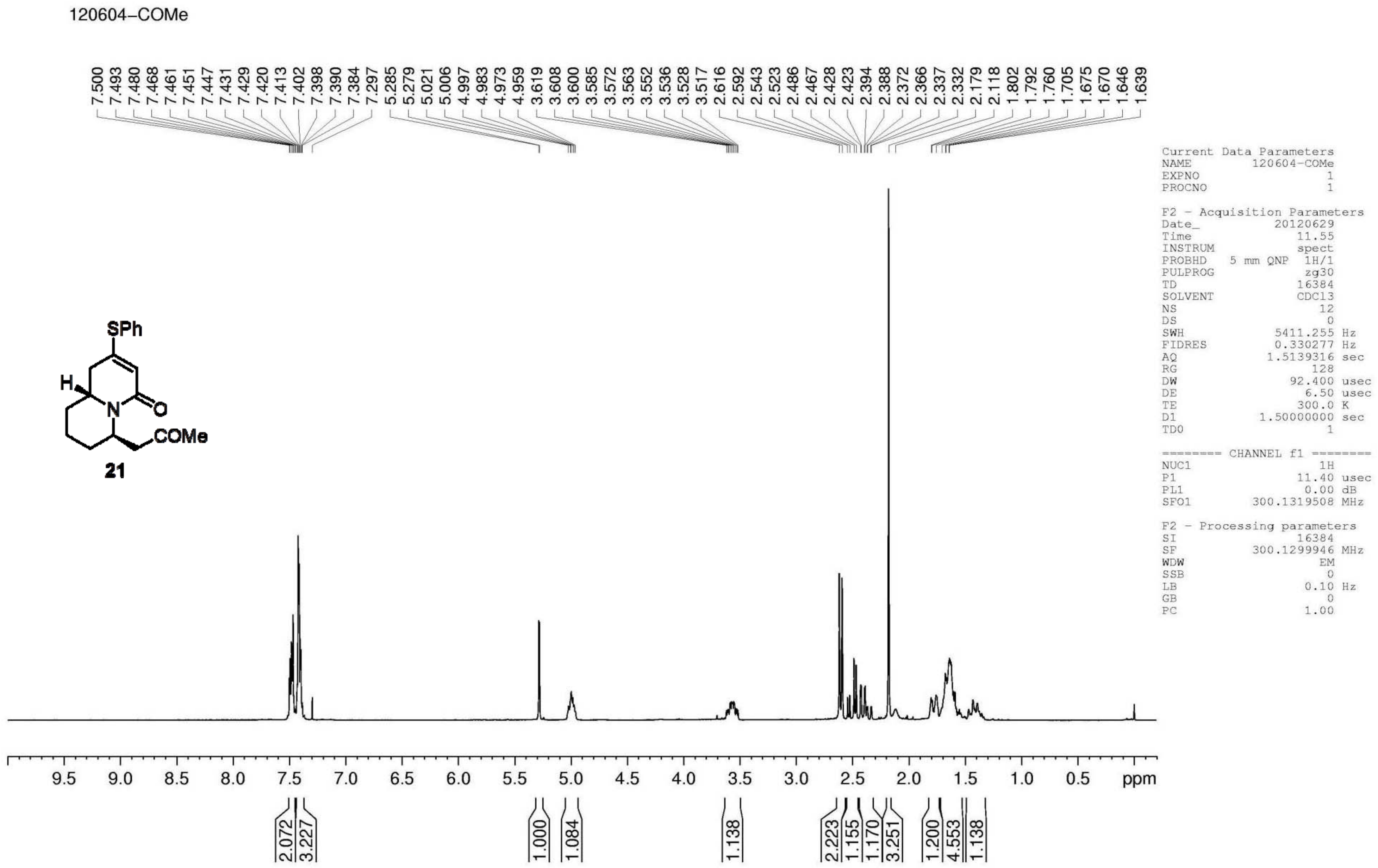
120604−COMe
7.500 7.493 7.480 7.468 7.461 7.451 7.447 7.431 7.429 7.420 7.413 7.402 7.398 7.390 7.384 7.297 5.285 5.279 5.021 5.006 4.997 4.983 4.973 4.959 3.619 3.608 3.600 3.585 3.572 3.563 3.552 3.536 3.528 3.517 2.616 2.592 2.543 2.523 2.486 2.467 2.428 2.423 2.394 2.388 2.372 2.366 2.337 2.332 2.179 2.118 1.802 1.792 1.760 1.705 1.675 1.670 1.646 1.639
SPh
H
N
O
COMe
21
Current Data Parameters
NAME 120604-COMe
EXPNO 1
PROCNO 1
F2 - Acquisition Parameters
Date_ 20120629
Time 11.55
INSTRUM spect
PROBHD 5 mm QNP 1H/1
PULPROG zg30
TD 16384
SOLVENT CDCl3
NS 12
DS 0
SWH 5411.255 Hz
FIDRES 0.330277 Hz
AQ 1.5139316 sec
RG 128
DW 92.400 usec
DE 6.50 usec
TE 300.0 K
D1 1.50000000 sec
TD0 1
======== CHANNEL f1 ========
NUC1 1H
P1 11.40 usec
PL1 0.00 dB
SFO1 300.1319508 MHz
F2 - Processing parameters
SI 16384
SF 300.1299946 MHz
WDW EM
SSB 0
LB 0.10 Hz
GB 0
PC 1.00
9.5 9.0 8.5 8.0 7.5 7.0 6.5 6.0 5.5 5.0 4.5 4.0 3.5 3.0 2.5 2.0 1.5 1.0 0.5 ppm
2.072 3.227 1.000 1.084 1.138 2.223 1.155 1.170 3.251 1.200 4.553 1.138

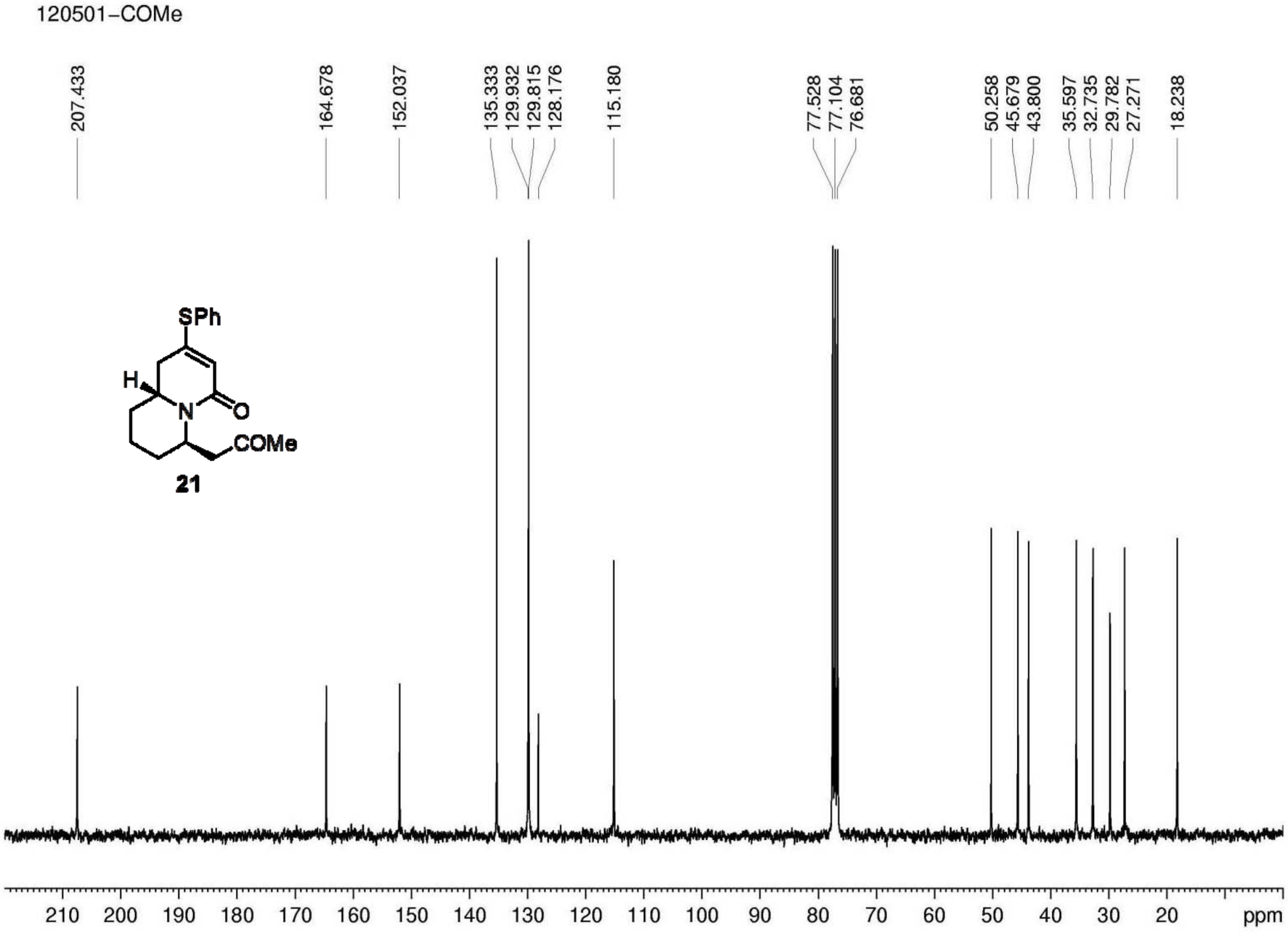


```
Current Data Parameters
NAME        120501-COMe
EXPNO                 2
PROCNO                1

F2 - Acquisition Parameters
Date_          20120502
Time               9.37
INSTRUM           spect
PROBHD   5 mm QNP  1H/1
PULPROG          zgpg30
TD                65536
SOLVENT           CDCl3
NS                  640
DS                    0
SWH          19267.822 Hz
FIDRES        0.294004 Hz
AQ           1.7007092 sec
RG             14596.5
DW              25.950 usec
DE                6.50 usec
TE               300.0 K
D1          2.00000000 sec
D11         0.03000000 sec
TD0                   1

======== CHANNEL f1 ========
NUC1                13C
P1                 9.50 usec
PL1               -1.50 dB
SFO1         75.4771825 MHz

======== CHANNEL f2 ========
CPDPRG2         waltz16
NUC2                 1H
PCPD2             90.00 usec
PL2                0.00 dB
PL12              18.00 dB
PL13              21.00 dB
SFO2        300.1313506 MHz

F2 - Processing parameters
SI                32768
SF           75.4677477 MHz
WDW                  EM
SSB                   0
LB                 3.00 Hz
GB                    0
PC                 1.00
```
